# Author Correction: A new ape from Türkiye and the radiation of late Miocene hominines

**DOI:** 10.1038/s42003-023-05358-0

**Published:** 2023-09-26

**Authors:** Ayla Sevim-Erol, David R. Begun, Alper Yavuz, Erhan Tarhan, Çilem Sönmez Sözer, Serdar Mayda, Lars W. van den Hoek Ostende, Robert M. G. Martin, M. Cihat Alçiçek

**Affiliations:** 1 Ankara University, Faculty of Languages History and Geography, Department of Anthropology, Ankara, Türkiye; 2 Department of Anthropology, University of Toronto, Toronto, ON Canada; 3 Mehmet Akif Ersoy University of Science and Letters, Department of Anthropology, Burdur, Türkiye; 4 Hitit University Faculty of Science and Letters, Department of Anthropology, Çorum, Türkiye; 5 Ege University Fakulty of Science, Department of Biology, İzmir, Türkiye; 6 Naturalis Biodiversity Center, Leiden, The Netherlands; 7 Pamukkale University, Department of Geology, 20070 Denizli, Türkiye

**Keywords:** Anthropology, Palaeontology

Correction to: *Communications Biology* 10.1038/s42003-023-05210-5, published online 23 August 2023.

In the original version of the Article, two authors and their affiliations were omitted.

In the original version of the Article, author first names were not provided for all authors.

The corrected author list and affiliations are provided below. This has now been corrected in the HTML and PDF versions of the article.

Ayla Sevim-Erol^1^, David R. Begun^2^, Alper Yavuz^3^, Erhan Tarhan^4^, Çilem Sönmez Sözer^1^, Serdar Mayda^5^, Lars W. van den Hoek Ostende^6^, Robert M. G. Martin^2^ & M. Cihat Alçiçek^7^

1 Ankara University, Faculty of Languages History and Geography, Department of Anthropology, Ankara, Türkiye

2 Department of Anthropology, University of Toronto, Toronto, ON, Canada

3 Mehmet Akif Ersoy University of Science and Letters, Department of Anthropology, Burdur, Türkiye

4 Hitit University Faculty of Science and Letters, Department of Anthropology, Çorum, Türkiye

5 Ege University Fakulty of Science, Department of Biology, İzmir, Türkiye

6 Naturalis Biodiversity Center, Leiden, The Netherlands

7 Pamukkale University, Department of Geology, 20070 Denizli, Türkiye

The Author Contributions section should read “A.S.E. is the PR of the project, secured funding for excavations and lab analysis and directed excavations. A.S.E. and D.R.B. are responsible for data curation, acquired funding and supervised research. D.R.B. conceptualized the analysis, conducted the investigation, developed the methodology, prepared the original draft and all subsequent versions. C.S.S., S.M., L.W.vdH. and C.A. contributed their analysis of geological and paleontological results. R.M.G.M. was responsible for the segmentation and analysis of the scans of the mandible. A.Y and E.T. participated in the excavations.” This has now been corrected in the HTML and PDF versions of the article.

