# Supplementary material for: A new ape from Türkiye and the radiation of late Miocene hominines
Source: Commun Biol. 2023 Aug 23;6:842. doi: 10.1038/s42003-023-05210-5 (PMC10447513; doi:10.1038/s42003-023-05210-5)
Supplement: Supplementary file 2 — Supplementary Information [file 42003_2023_5210_MOESM2_ESM.pdf]

# Supplementary Information for

**Title: A new ape from Türkiye and the radiation of late Miocene hominines**

**Authors:** A. Sevim Erol[1*], D.R. Begun[2*], Ç. Sönmez Sözer[1], S. Mayda[3], L.W. van den Hoek Ostende[4], R.M.G. Martin[2], M. Cihat Alçiçek[5]

[1]Ankara University, Faculty of Languages History and Geography, Department of Anthropology, Ankara, Türkiye

[2]Department of Anthropology, University of Toronto

[3] Ege University Fakulty of Science, Department of Biology, İzmir, Türkiye.

4 Naturalis Biodiversity Center, Leiden, The Netherlands

[5]Pamukkale University, Department of Geology, 20070, Denizli, Türkiye

*Corresponding authors: David R. Begun (david.begun@utoronto.ca) and Alya Sevim Erol (aylasevimerol@gmail.com)

**This file includes:**


| Headings | Figures and tables | Pages |
|---|---|---|
| Supplementary Note 1 | Institutions and samples | 2 |
| Supplementary Note 2 | Descriptions and measurements | 3-21 |
| Supplementary Note 3 | Differential diagnosis | 22-24 |
| Supplementary Note 4 | Quantitative analyses | 25-42 |
| Supplementary Note 5 | Phylogenetic analysis | 43-51 |
| Supplementary Note 6 | Historical, geological and taxonomic background | 52-63 |
| Supplementary References | | 64-70 |

**Supplementary Note 1: Samples and Institutions**

| Abbreviation | Institution | Taxa |
|---|---|---|
| AUDA | Ankara University Department of Anthropology | *Anadoluvius* |
| AMNH | American Museum of Natural History | *Sivapithecus*/extant catarrhines |
| MCZ | Museum of Comparative Zoology, Harvard University | Extant catarrhines |
| Harvard | Department of Anthropology, Harvard University | *Sivapithecus* |
| NMNH | National Museum of Natural History (Smithsonian Institution) | Extant catarrhines |
| CMNH | Cleveland Museum of Natural History | Extant catarrhines |
| IHO | Institute of Human Origins (Arizona State University) | *Australopithecus* |
| UCB | University of California Berkeley | *Ardipithecus* |
| UPENN | University of Pennsylvania | Extant catarrhines |
| ANS | Academy of Natural Sciences (Philadelphia) | Extant catarrhines |
| YP | Yale Peabody Museum, Yale University | *Sivapithecus* |
| MRC | Musée Royal de l'Afrique Centrale (Tervuren, Belgium) | Extant catarrhines |
| NHM | Natural History Museum (London) | *Griphopithecus, Proconsul* |
| NMK | National Museums of Kenya | Miocene apes/*Australopithecus* |
| ENM | Ethiopian National Museum | *Ardipithecus/Australopithecus* |
| IVPP | Institute of Vertebrate Paleontology and Paleoanthropology (Beijing) | *Lufengpithecus* |
| MTA | Maden Tetkik ve Arama Museum of Natural History (Ankara) | *Ankarapithecus/Griphopithecus* |

Supplementary Table 1. Institutions visited and collections analyzed for this research.

| Taxon | Collection | Reference |
|---|---|---|
| *Anadoluvius* | AUDA | This paper |
| *Ekembo* | KNM,NHM | 1,2 |
| *Equatorius* | KNM | 3,4 |
| *Nacholapithecus* | KNM | 4 |
| *Kenyapithecus* | KNM | 1 |
| *Nakalipithecus* | KNM | 5 |
| *Ouranopithecus* | AUT | 6,7 |
| *Rudapithecus* | NHMH, GMH | 8 |
| *Hispanopithecus* | ICP | 9 |
| *Pierolapithecus* | ICP | 9 |
| *Anoiapithecus* | ICP | 9 |
| *Dryopithecus* | ICP, MNHN, MHNB | 9,10 |
| *Ankarapithecus* | MTA[1] | 11,12 |
| *Sivapithecus* | YPM, AMNH, HAR | 13,14 |
| *Ardipithecus* | NME | 15 |
| *Sahelanthropus* | UP (Casts) | 16 |
| *Samburupithecus* | KNM | 17 |
| *Chororapithecus* | NME | 18 |
| *Graecopithecus* | UT | 19 |
| *Orrorin* | MNHN (casts) | 20 |

Supplementary Table 2.: Fossil samples included in this analysis. AUD=Ankara University Department of Anthropology; KNM=National Museums of Kenya; NHM=Natural History Museum (London); AUT=Aristotle University Thessaloniki; NHMH=Natural History Museum of Hungary; GMH Geological Museum of the Hungarian Mining and Geological Survey; IPC=Institut Català de Paleontologia Miquel Crusafont ; MNHN=Muséum Nationale d’Histoire Naturelle; MHNB= Muséum d’Histoire Naturelle de Bordeaux; MTA=Maden Tetkik ve Arama Museum of Natural History (Ankara); YPM=Yale Peabody Museum; AMNH=American Museum of Natural History; HAR=Harvard Department of Anthropology; ENM=Ethiopia National Museum; UP=University of Poitiers; UT=University of Tübingen; . Data collected from all published original specimens in these collections. Hypodigms listed in the references cited.

Notes: 1. Data collected from the type and male palate supplemented by published data.

**Supplementary Note 2**

*Descriptions*

CO-205, the holotype, (Supplementary figure 1), represented by the two palatal fragments and two isolated teeth, was partially described in (21), though their descriptions are largely limited to the dentition. It is larger than CO-2100/2800 (Supplementary figures 2-3). The description of the larger mandible, CO-305 is also limited to the teeth. Other specimens figured in (21), CO-300 and CO-710 (Supplementary figure 4), were not included in their descriptions and will be described here along with the face and associated $RI^1$ (CO-2100 & CO-2800). We also supplement the descriptions of CO-205 and CO-305 with anatomical details not included in the initial descriptions.

**CO-205** The reconstruction of CO-205 figured in (21) differs significantly from the current reconstruction (Supplementary figure 1). The holotype was molded and cast by DRB in 2000. High resolution RTV silicon was used but we do not have records of the brand. Casts were produced using ultrafine dental stone. (21) provide an image of CO-208 (figure 1) partially restored. DRB, who is not an author of (21), cleaned and restored this specimen in 2000. The images in (21) show a specimen that had sustained some damage after preparation [see also (10).] A piece of the left premaxilla including the partial alveolus for the left $I^1$ was detached and re-glued incorrectly, resulting in displacement to the right of the alveolus and both incisors. These errors have been corrected on the original specimen and the new restoration. In (21) the anterior portion of the palate and the incisors are incorrectly positioned relative to the midline (Supplementary Figure 1). This is perhaps what led (21) to conclude that the anterior portion of the palate was narrow. In fact, though the palate widens

slightly distally it is not narrow anteriorly, matching a compatible mandible of *Ouranopithecus* (RPl 89.) CO-205 is described as distorted (21). There is actually very little distortion although, as noted, the specimen is fragmented (which probably limited the distortion.) Its restoration, unlike CO-2800, was relatively uncomplicated (see above.) The palate is shallow anteriorly and deepens posteriorly so that opposite the level of the $M^3$s (only the left one is preserved) it is quite deep. The position and orientation of the incisors are clear from the connection between the premaxillary fragment with the $I^1$ alveolus and the premaxillary fragment attached to the right canine. This confirms, as described in (21), that the premaxilla was relatively short and vertically oriented. The incisors are in the transverse plane of the canines. The lingual surface of the $I^2$ is level with this plane (estimated) while the lingual surface of the $I^1$ is slightly anterior to it. We can also confirm that the pre-canine diastema is small and the palatine process thin (21).

The remaining description of CO-205 expands on that provided in (21). The right palatine surface of the premaxilla preserves a trace of the intermaxillary suture and the anterior and lateral margins of the right incisive foramen (Supplementary Figure 1). The canine prominence on the right side and the left upper central incisor serve to delimit portions of the premaxillary alveolar process, which is relatively short and vertically oriented. With the incisive edges of the incisors positioned in the occlusal plane with the buccal cusp tips of the postcanine dentition, as they always are in hominids, the tips of the incisor roots are well superior to the palatine process of the maxilla, resulting in a strongly developed step. The position of the base of the nasal aperture is consistent in hominids, mid-way between the canines. Positioning the base of the nasal aperture in CO-205 results in a relatively short nasoalveolar clivus most like that of *Ouranopithecus, Rudapithecus, Dryopithecus, Anoiapithecus, Pierolapithecus* and *Gorilla*.

Most of the alveolar processes on both sides are preserved. There is some displacement of teeth on the right side, but the left tooth row is undistorted. The increase in depth is clear from the alveolar process on the left side from the canine through the $M^2$. The alveolar process for the left $M^3$ is minimally preserved, but sufficient to accurately position the $M^3$. Lingually the alveolar processes are vertical, less inclined than in *Ankarapithecus* and more like *Ouranopithecus*.

The superior portions of the alveolar processes are preserved on both sides of the maxilla, but more of the alveolar bone is present on the left side. The maxillary sinus reaches the canine alveolus below the level of the root, which overhangs the sinus anteriorly. The lingual root of the $P^3$ pierces the floor of the sinus. More posteriorly the floor invaginates the alveolar process, especially deeply between $M^1$-$M^2$. The roots of the canines are more vertically oriented compared with *Ouranopithecus* and implanted in alignment with the postcanine tooth row (they are medially inclined in *Ouranopithecus*.) Suplementary Figure 5 is a comparison among male palates of *Ouranopithecus* and *Anadoluvius*.

**CO-300/305** (Supplementary Figure 4). CO 305 is a partial mandible preserving portions of the left and right alveolar processes. The base is absent. The symphysis includes the roots of the incisors. The left alveolar process of the CO-305 mandible is well preserved from $P_3$ to $M_1$ while the right corpus lacks most of the buccal cortical bone. There is a crack between the $I_2$ alveolus and the canine on the right side, but the symphysis is in close to proper position relative to the right corpus. The lingual surface of the symphysis is similar in inclination to male *Ouranopithecus*, more inclined than in female *Ouranopithecus*. The right canine is pushed into the alveolus. The right $P_3$ to $M_1$

are in proper alignment. The symphysis is damaged. It lacks the base and the alveolar portion is crushed on the left side but the alveolar portion is in its original position on the right side. The left corpus fragment includes the alveolar process and $P_3$-$M_1$. It is pressed against the crushed symphysis and rotated inferiorly relative to the right alveolar process. CO 300 is a right $M_2$ with an interproximal facet that matches the right $M_1$ of CO 305. It is a good match in terms of overall size and degree of wear and is very likely from the same individual.

The shape of the symphysis in cross section cannot be determined. The right corpus retains most of the alveolar process lingually between the canine to distal to $M_1$. About 18.5 mm are preserved below P4 with no sign of the base, suggesting that the corpus may have been relatively tall, as in *Ouranopithecus* males. On the left side the alveolar process of the corpus is intact between $P_3$ and $M_1$. The surface of the alveolar process lingually is flush with or slightly buccal to the lingual surfaces of the $M_1$'s, as in NKT 21 and *Graecopithecus.* The mental foramen is below $P_3$-$P_4$. There is a broad concavity between mid $P_3$ to mid $M_1$, posterior to which is the anterior inception of the mandibular eminence.

The right canine is well preserved but pushed into its alveolus, making it difficult to visualize on the original specimen or casts. However, we were able to segment and virtually extract the canine using µCT scan data (Supplementary Figure 10; see Methods). It is tall crowned, narrow with mesial and distal edges meeting at a strongly acute angle. The cervix is flat (unflared). These attributes indicate that the mandible is most likely male, as concluded by (21). The cervical dimensions of the canines are small relative to the dimensions of the mandible and the postcanine teeth.

The premolars and $M_1$ are well preserved on both sides. The $P_3$ is triangular with strongly developed cingula running from the mesial end of the preprotocristid (mesiobuccal) lingually to the distal end of the postprotocristid (distobuccal.) The mesial and distal fovea are compressed and the protolophid is broad and rounded. The cervix is flared buccally and moderately expanded onto the mesiobuccal root. The $P_4$ is relatively long with an elongated talonid and equally sized mesial cusps. The mesial fovea is small and the talonid basin is shifted lingually. The crown is strongly flared buccally and relatively tall crowned, as is the case for all the postcanine teeth. The $M_1$ is longer than broad with broad, flat buccal cusps, a shallow talonid basin, slit-like mesial fovea and a small, deep, lingually placed distal fovea. The right $M_1$ has a malformation in the region of the entoconid, which is reduced and replaced in part with two deep pits lingually and distally. On the left $M_1$ the hypoconulid is divided into two small cusps. CO-300 is a right M2 morphologically similar to the $M_1$ but with a well-developed tuberculum sextum in place of the distal fovea.

<u>**CO-710**</u> (Supplementatry Figure 4) A right $P_3$ to $M_2$ held together with small fragments of the alveolar process. The lingual surface of the alveolar process is preserved to about the level of the root tips. Like CO-300, the molar lingual surfaces overhang the alveolar lingual surface, which is flush with the $P_4$ and lingual to the lingual margin of the P3. The teeth are strongly worn. The $P_3$ is more oval than in CO-305, with a shorter crown that is not expanded mesiobuccally onto the mesiobuccal root. This morphology along with the much smaller size of CO-710 compared with CO-305 lead us to conclude that it is female. The $P_3$ protolophid is thick and expanded lingually into a slightly developed metaconid. The mesiolingual corner is notched. The $P_4$ is similar to CO-305 in occlusal proportions, as are the molars, though the $M_2$ lacks the tuberculum sextum.

**CO-2100/2800** (Supplementary Figures 2-3). A partial cranium preserving the right maxillary alveolar process with canine to $M^2$, most of the right palatine process of the maxilla, a piece of the right premaxilla preserved slightly beyond the midline with the partial alveoli of the central incisors and right $I^2$, the left and right nasal margins and portions of the malar surfaces of the maxilla, most of both frontal processes of the maxilla and a nearly complete frontal bone. CO 2100, a right $I^1$, was found separately and is likely associated with CO-2800.

The premaxillary alveolar clivus is preserved from prosthion to the base of the nasal aperture on the right side. Distortion in the maxilla between the alveolar process and the nasal pillar was corrected, including the orientation of the premaxilla (see Methods for details). Alignment with the maxilla as well as a constraint imposed by the length of the $I_1$ root, positions the base of the nasal aperture superior to and overlapping the palatine process of the maxilla, producing a stepped subnasal fossa.

Areas of distortion that remain after reconstruction of CO-2100/2800 are apparent in figure 1. Most obvious is the right alveolar process, which is crushed and pushed superiorly and medially into the infratemporal fossa (lateral view.) Plastic deformation between the nasal pillar and the maxillary alveolar process shifts the nasal margin laterally, distorting the breadth of the nasal aperture (anterior view). The nasal aperture is further artificially enlarged by distortion between the maxillary and zygomatic portions of the inferior orbital margin on the left side. These distortions have been corrected in the reconstruction, which was informed by preserved contours, matching conjoining breaks, and comparisons with *Ouranopithecus* and CO-205 (Supplementary Figure 2).

As noted in the methods section on reconstructing the specimens, the midline of the palate is preserved on the right palatine process and the right premaxillary fragment. The

palate is broad anteriorly and slightly divergent posteriorly, as in CO-205. The premaxillary fragment cannot be attached directly to the right maxilla, making it difficult to determine the exact length and orientation of the nasoalveolar process. The base and margins of the nasal aperture are partly preserved. The base is flat and the margins are sharp superiorly and proximally projecting. The infraorbital surface is anterolaterally facing. The lateral orbital pillars are thick, and the lateral orbital margin is rounded. On the zygomatic process of the frontal bone just superior to the fronto-zygomatic suture on both sides there are prominent flattened posteriorly oriented flanges at the anterior ends of the temporal lines. The interorbital space is broad, convex transversely and concave sagittally. A sinus extends from the frontal squama to below nasion, suggestive of an ethmoidally derived frontal sinus. The nasal processes of the frontal bone are well preserved as are portions of the nasal bones, which are relatively broad. Based on this morphology and the position of the nasal processes of the maxilla it is likely that the nasal bones were relatively short. The superior portion of the lacrimal fossa is preserved, indicating that it was internal to the orbit.

The temporal lines or crests are strongly developed from the anterior temporal flanges to the posterior end of the frontal bone, which is preserved just anterior to the coronal suture. The temporal crests are strongly convergent, well-developed and probably would have met to form a sagittal crest posterior to bregma. The frontal squama is broad and biconvex along most of its surface. About 15 mm anterior to the distal break the space between the temporal crests becomes transversely concave. The frontal squama is oriented at roughly 50 degrees relative to a vertically oriented lateral orbital pillar. A portion of the temporal fossa is preserved on the right side. It is broad transversely, suggestive along with the strongly developed temporal crests of a large, well developed temporalis muscle. The

superior orbital margin is well preserved and sharply delineated. There is a slight projection anteriorly but no evidence of a supratoral sulcus or circumorbital rims.

Portions of the rostral and superior regions of the frontal endocranial surfaces are well-preserved. The frontal crest is preserved over a short distance superior to the orbital margins, fading distally but traceable to the break. The preserved portions of the frontal lobe surfaces are asymmetric, with a right petalia, being broader and more rounded, while the supero-rostral surface of the left frontal lobe is flatter. A well-marked crest, probably corresponding to the superior frontal sulcus, is discernable on the left side. The dimensions of the frontal portion of the cranial vault are comparable with *Gorilla*, suggestive of comparably sized frontal lobes.

The upper central incisor (CO 2100) is complete from the incisive edge to the root tip. The root is long and mildly curved in the sagittal plane. The crown, which is minimally worn, is lower crowned than in *Ouranopithecus*, while the cervix is expanded lingually with a prominent bulge. The lingual bulge is continuous with a well-defined lingual pillar, which fades about halfway to the cervix. The lateral marginal ridge is thick, rounded and straight, fading into the lingual surface of the incisor at the same level as the lingual pillar. The mesial marginal ridge is less pronounced, possibly due to wear. The tooth flares mildly toward the cervix. The canine of CO 2800 is smaller in cervical dimensions and lower crowned than CO 205. We interpret this difference to indicate that CO 205 is a male and CO 2800 a female. The canine of CO 2800 is triangular in cross section, with a shallow, narrow mesial groove and a prominent distolingual bulge, resembling *Ouranopithecus* in this regard. The bulge is separated from the remaining lingual crown face by a centralized pit and does not arise from the cingulum as a pronounced tubercle, as in *Nakalipithecus*. The mesial and distal marginal

ridges are long relative to crown height, resulting in relatively low crown shoulders. The cervix is smooth, lacking any sign of a cingulum. As in CO 205 the premolars of CO 2800 are homomorphic, both having nearly equally well developed lingual and buccal cusps. The $P^3$ is longer than the $P^4$, but both relatively broad, with broad, rounded cusps, lacking a crista between the two. The mesial and distal marginal ridges are poorly defined, as are the fovea. The molars are heavily worn but remain relatively tall crowned. The enamel of the $M^1$ is worn off the lingual cusps near to their bases, with only a strip of enamel remaining lingually. Most of the paracone is also worn to the dentine. The $M^1$ is close in size to the $M^2$. The cusps are nearly equal in size within each tooth, although the metacone is somewhat smaller than the other cusps in the $M^2$. The distal cusps are aligned with the mesial cusps and not offset (lingually displaced) as in most hominoid upper molars. Comparisons with other fossil apes are provided in the differential diagnosis (below).

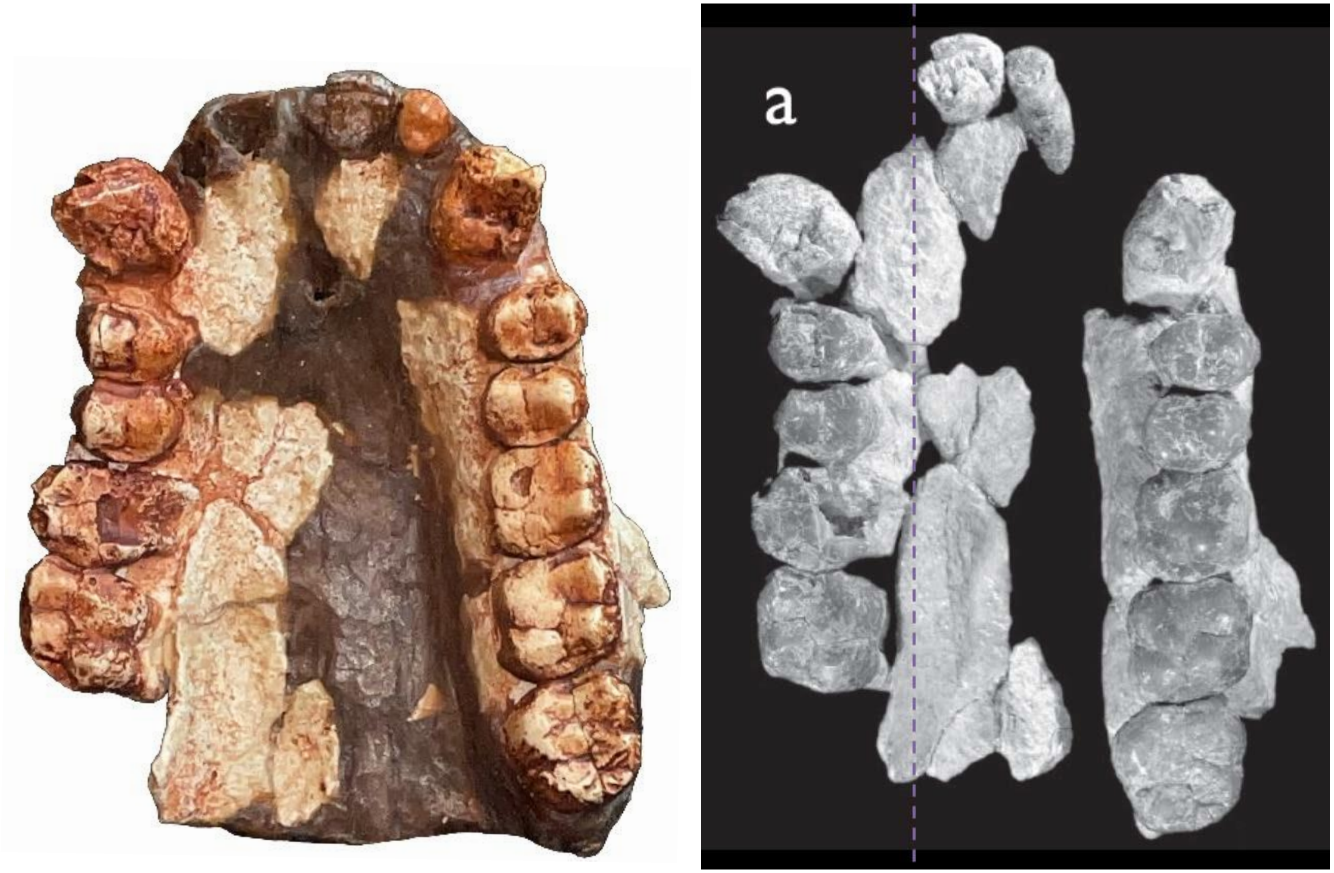


Supplementary Figure 1. Reconstruction of the *Anadoluvius* holotype (CO-205). Right, from (21). Note the displacement of the incisors to the right of the midline. Parts of the palatine process that include portions of the canine and incisor alveoli are misaligned. Left, cast of CO-205 restored to its original reconstruction. The cast was produced prior to the photo from (21). The original fossil lost some of the premaxillary palatine process, including the lingual margin of the right $I^1$ alveolus, prior to its arrival in its current repository. In the restoration the midline is preserved on the upper central incisor and along various points of the intermaxillary suture. Note the position of the incisors relative to the canines in the restoration. These are correctly described in (21) as in alignment with the canines, though they are positioned well anterior to the canines in the figure from that publication. Scale = 10 mm.

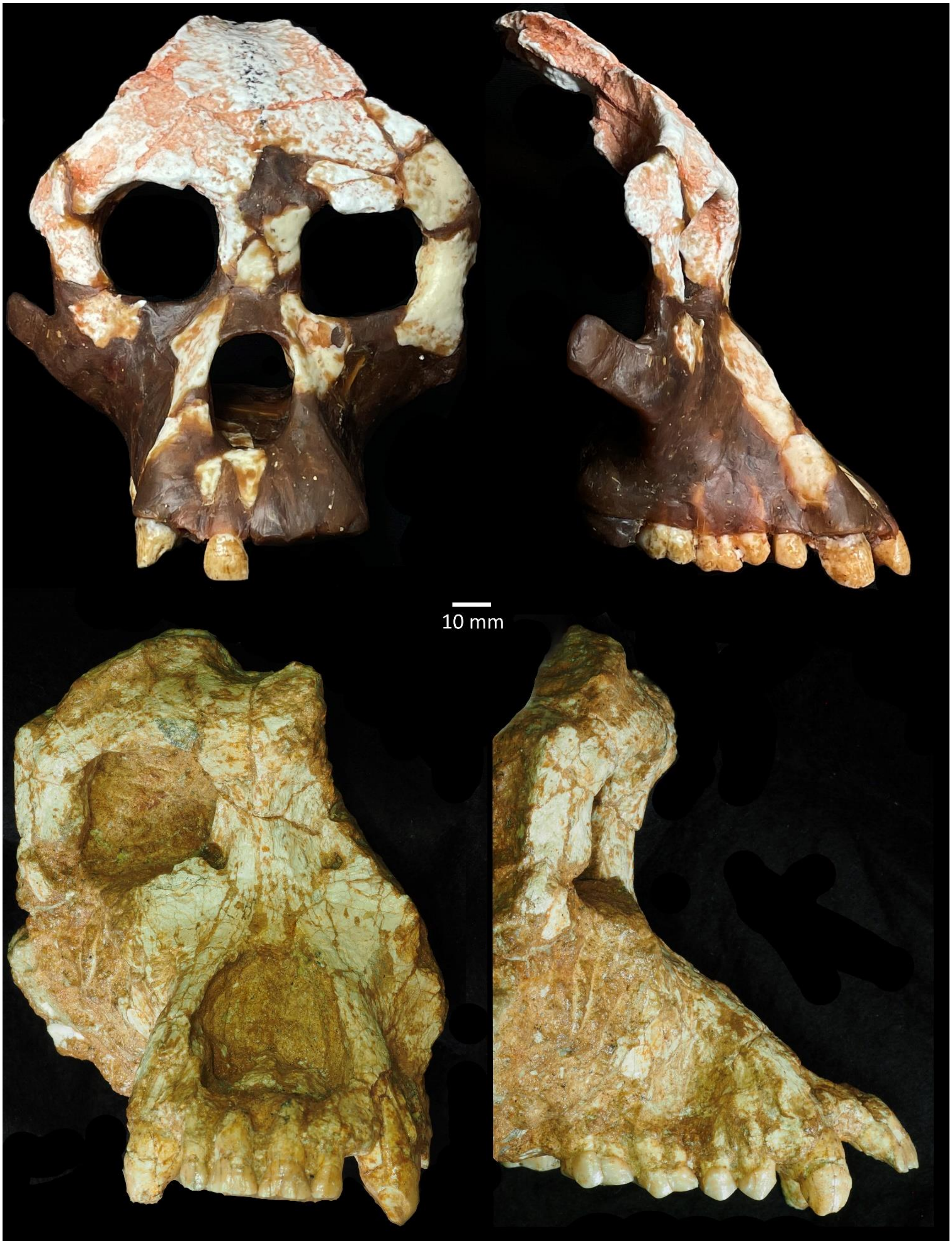
10 mm

Supplementary Figure 2. Comparison between the reconstruction of the female *Anadoluvius* CO-2100/2800 (upper row) and the male *Ouranopithecus* XIR-1 (22) (lower row) in anterior (left) and lateral (right) views. For the reconstruction of CO-2100/2800, high resolution casts of elements separated during preparation of the original were assembled according to congruent joint surfaces and symmetry. On the right side the cast of the maxillary body including the nasal margin was cut from the maxillary alveolar process, which is distorted. The maxillary alveolar process was cut into three pieces so that the teeth could be aligned close to proper anatomical position, though some residual distortion remains. The right lateral orbital pillar was detached from the frontal and the small portion of the zygomatic bone near the inferolateral orbital margin so that they could be realigned. The interorbital fragments were also detached from one another and realigned in proper anatomical position. Compare with figure 2. Note the differences between CO-2100/2800 and XIR-1 (*Ouranopithecus macedoniensis* from Xirochori, Macedonia, Greece) in premaxillary orientation, incisor position relative to the canines, nasal aperture size and shape, maxillary breadth between the nasal aperture and orbits, interorbital breadth, supraorbital torus height and projection, supratoral groove and frontal orientation. Reconstruction by DRB.

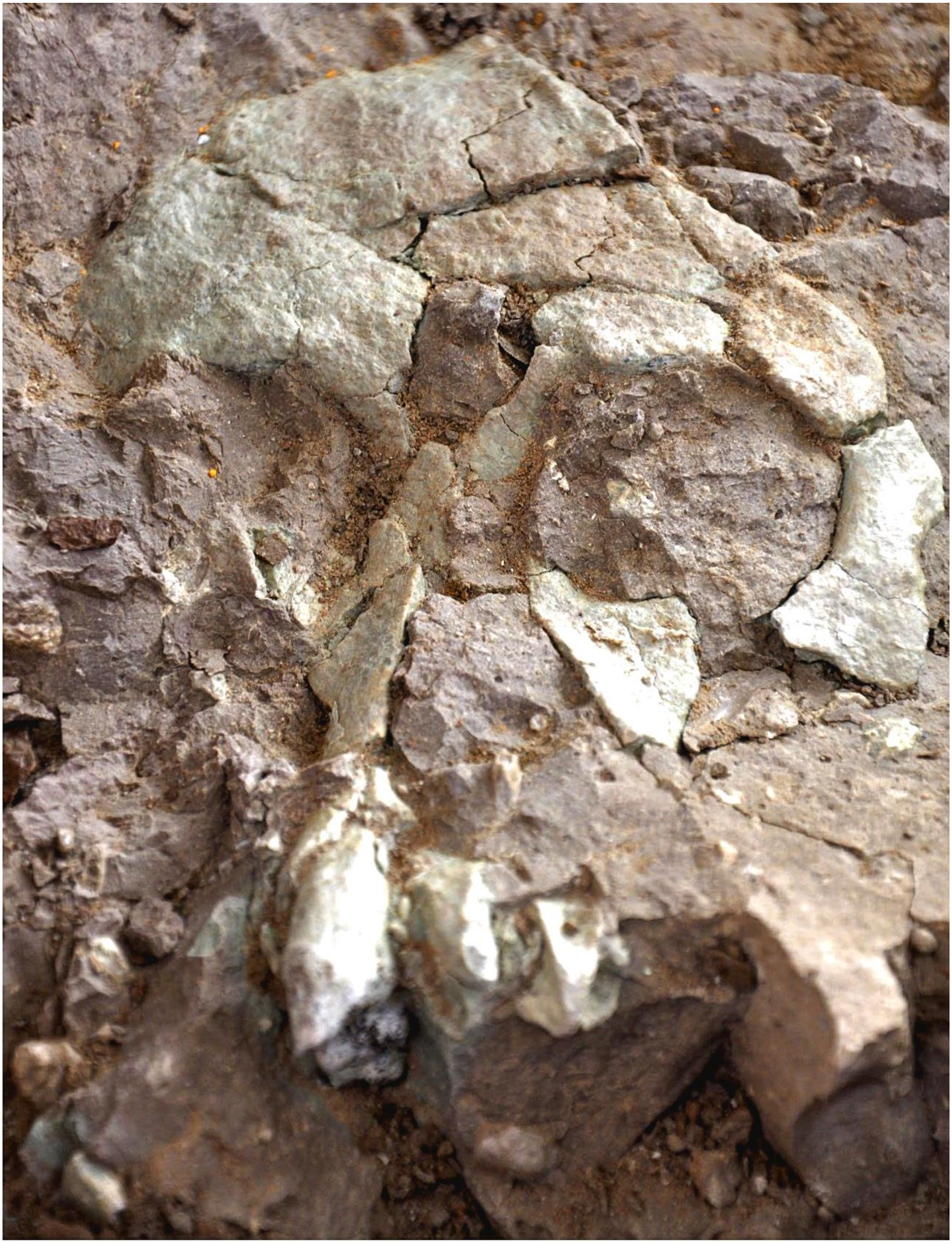

Supplementary Figure 3: CO-2800 *in situ*.

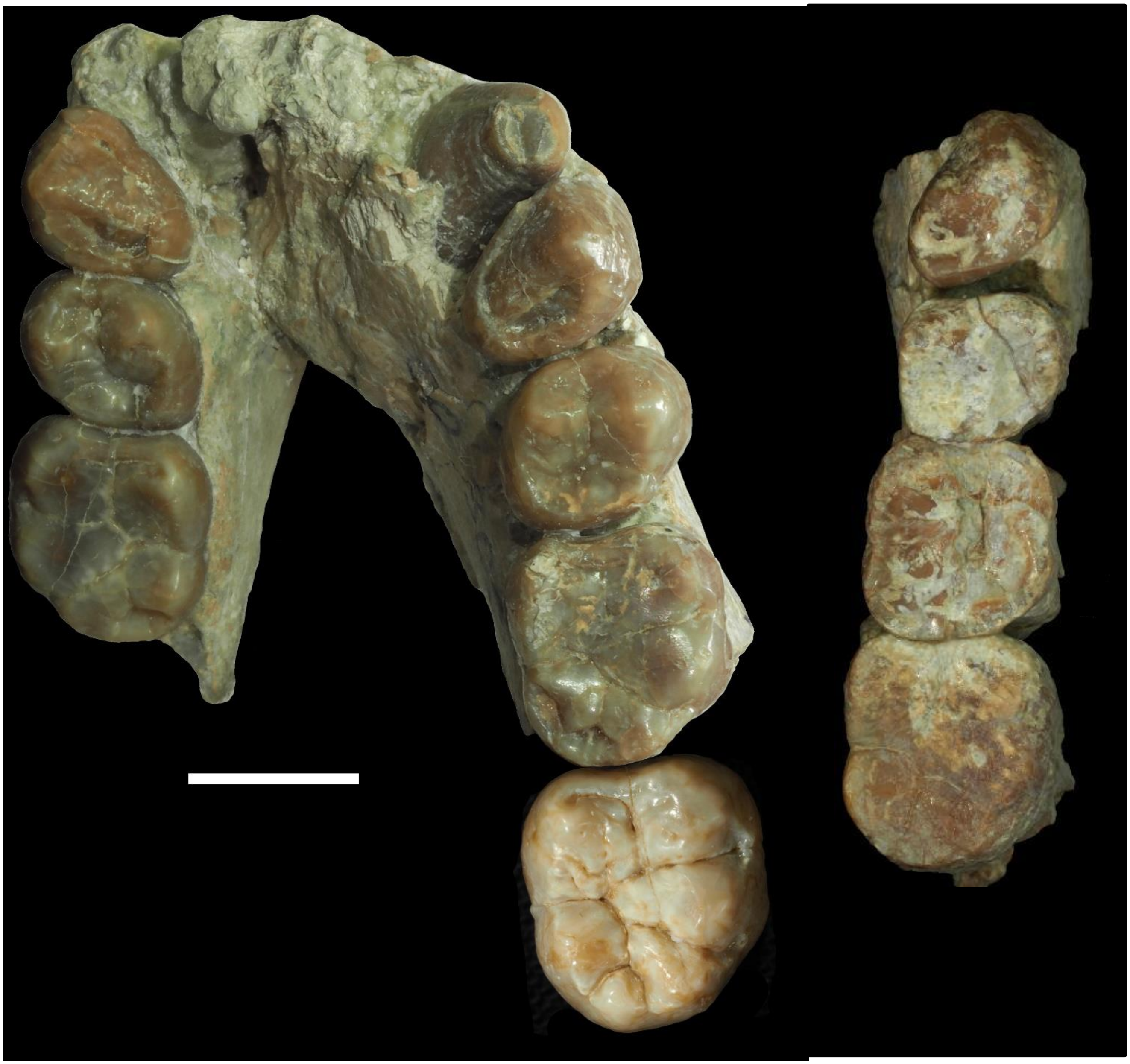

Supplementary Figure 4. CO-305 (left) and CO 710 (right) in occlusal view. Scale = 10 mm

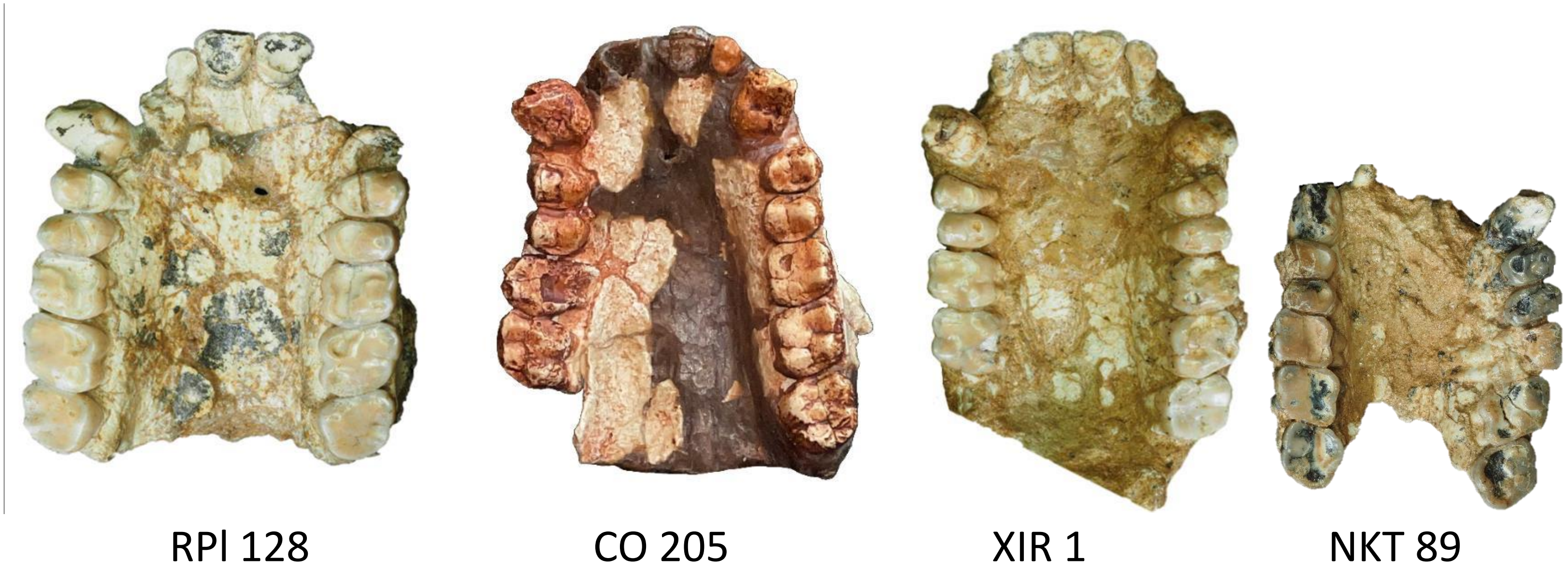


Supplementary Figure 5. Occlusal views of male *Ouranopithecus* (fossils) and *Anadoluvius* (cast) palates. Note in *Anadoluvius* differences from *Ouranopithecus* in the position of the incisors, the length and homodonty of the premolars, relative molar length and the breadth of the canines. *Anadoluvius* more closely resembles NKT 89 in these attributes.

| Lower | CO-300 | CO-305 | CO-710 | Upper | CO-205 | CO-2800 | CO-2100 |
|---|---|---|---|---|---|---|---|
| rc max | | 12.2 | | RI1 L | | | 10.7 |
| rc perp | | 8.7 | | RI1 B | | | 9.5 |
| rc Lab Ht | | 20 | | RI2 L | | | |
| rp3 max | | 12.6 | 10.4 | RI2 B | | | |
| rp3 perp | | 9 | 7.3 | RC MAX | 15.3 | 13.2 | |
| rp3 L | | 9.4 | 8.8 | RC PERP | 15.4 | 13.8 | |
| rp3 B | | 11.4 | 9.6 | RC Lab Ht | 21.1 | | |
| rp4 L | | 9.8 | 7.9 | RC ling Ht | 20.2 | | |
| rp4 B | | 11.9 | 9.6 | RC root L mesial | 30.5 | | |
| rm1 L | | 14.1 | 10.9 | RP3 L | 10 | 9.2 | |
| rm1 B | | 13.1 | 10.7 | RP3 B | 14.1 | 13.1 | |
| rm2 L | 15.8 | | 12.7 | RP4 L | 10 | 9.6 | |
| rm2 B | 14.4 | | 11.7 | RP4 B | 14.3 | 13.8 | |
| lp3 max | | 13.3 | | RM1 L | 14 | 13.2 | |
| lp3 perp | | 8.8 | | RM1 B | | 14.5 | |
| lp3 L | | 9.7 | | RM2 L | 15.8 | 13.8 | |
| lp3 B | | 12.2 | | RM2 B | 16.2 | 15.8 | |
| lp4 L | | 9.6 | | LI1 L | 11.6 | | |
| lp4 B | | 12.3 | | LI1 B | 10.2 | | |
| lm1 L | | 13.5 | | LI2 L | 6.8 | | |
| lm1 B | | 13 | | LI2 B | 7.6 | | |
| | | | | LC MAX | 14.7 | | |
| | | | | LC PERP | 15.6 | | |
| | | | | LP3 L | 10.1 | | |
| | | | | LP3 B | 14.3 | | |
| | | | | LP4 L | 10.1 | | |
| | | | | LP4 B | 14.7 | | |
| | | | | LM1 L | 14.2 | | |
| | | | | LM1 B | 15.3 | | |
| | | | | LM2 L | 15.8 | | |
| | | | | LM2 B | 16.4 | | |
| | | | | LM3 L | 17 | | |
| | | | | LM3 B | 18.1 | | |

**Supplementary Table 3**. Upper and lower dental measurements (in mm.) Abbreviations: lower teeth in lower case, upper teeth in upper case; L=length, B=breadth, max=maximum length, perp=perpendicular breadth, Lab HT=labial crown height, ling HT=lingual crown height, root L mesial=mesial root length.

| Cranial and mandibular dimensions | CO-305 | CO-205 | CO-2800 | Measurement definitions |
|---|---|---|---|---|
| Orbital breadth | | | 34.5 | Maximum breadth between the medial and lateral orbital walls |
| Orbital height | | | 31 | Maximum height between the superior and inferior orbital walls |
| Interorbital breadth | | | 21 | Maximum distance between the orbits |
| Bi-orbital breadth | | | 115 | Distance between the lateral margins of the orbital pillars at mid-orbit |
| Postorbital breadth | | | 67.5 | Minimum distance between left and right temporal fossae |
| Glabella-prosthion chord | | | 87.5 | Distance from glabella to prosthion |
| Nasal aperture breadth | | | 22.5 | Maximum breadth of the nasal aperture |
| Nasal aperture height | | | 30 | Maximum height of the nasal aperture |
| Palatal length (I1-M2) | | 74 | 66.5 | Palatal length from Prosthion to the transverse between the distal M2 edge |
| Intercanine palatal breadth | | 31.5 | 30 | Breadth between the lingual margins of the canine alveoli |
| Palatal breadth at P3 | | 38.4 | 28 | Breadth between the lingual margins of the P3 alveoli |
| Palatal breadth at P4 | | 40 | 29 | Breadth between the lingual margins of the P4 alveoli |
| Palatal breadth at M1 | | 42 | 31.6 | Breadth between the lingual margins of the M1 alveoli |
| Palatal breadth at M2 | | 47 | 34 | Breadth between the lingual margins of the M2 alveoli |
| Palatal breadth at M3 | | | | Breadth between the lingual margins of the M3 alveoli |
| Mental foramen-alveolar margin | 16.4 | | | Minimum distance from the mid mental foramen to the alveolar margin |
| Mandibular breadth @ p4 | 14.5 | | | Breadth between the lingual margins of the p4 alveoli |
| Mandibular breadth @ p4-m1 | 15.6 | | | Breadth between the lingual alveolar margin between p4-m1 |
| Mandibular breadth @ m1 | 17.0 | | | Breadth between the lingual margins of the m1 alveoli |
| Mandibular breadth @ m1-m2 | 20 | | | Breadth between the lingual alveolar margin between m1-m2 |
| m1 distobuccal root length | 13.6 | | | Distance from the cervix to the root apex |
| p4 mesiobuccal root length | 15 | | | Distance from the cervix to the root apex |

**Supplementary Table 4: Cranial and mandibular measurements (in mm)**

## Supplementary Note 3

## Differential diagnosis

*Anadoluvius* differs from *Ouranopithecus* in having upper incisors positioned more in-line with the canines, relatively lower crowned but cervically enlarged, vertically implanted canines, homomorphic premolars, elongated premolars and molars, convex frontal squama with less pronounced torus, sharp superior orbital margins, absence of molar cingula and a different lower postcanine root/root canal morphology. *Anadoluvius* differs from *Graecopithecus* in having larger canines, smaller molars relative to mandibular corpus breadth, a more robust mandible, a longer symphysis-m1/m2 chord, and a relatively short $P_4$. *Anadoluvius* differs from *Samburupithecus* in its homomorphic premolars, the absence of upper molar lingual cingula, more rounded cusps and broader, shallower occlusal basins, and in its higher zygomatic root. *Anadoluvius* differs from *Chororapithecus* in having smaller $M^2$ and $M^3$ on average, broader $M^2$ and a male lower canine with a more compressed crown and root, more prominent lingual bulge, and flatter cervical lines lingually and buccally.

*Anadoluvius* differs from *Nakalipithecus* in having mediolaterally narrower, labiolingually thicker $I^1$, with more symmetrical crown, broader lingual bulge and thicker marginal ridges. The female upper canine of *Nakalipithecus* is much smaller than in *Anadoluvius*, which lacks the pronounced, compressed lingual tubercle and cingulum of the former. The mesial groove of the upper canines of *Anadoluvius* is less well-defined, and the lingual surface is broadly biconvex, unlike the concave surface in *Nakalipithecus. Nakalipithecus* lacks the homomorphic upper premolars of *Anadoluvius*, having a more typical triangular $P^3$. In *Nakalipithecus* the $P^4$ has taller, pointier (buccolingually compressed) cusps, broader basins and strongly developed mesial and distal marginal ridges. The $P_3$ of *Nakalipithecus* (probably female) is more obliquely elongated than the female *Anadoluvius* specimen (CO-710), with deeper fovea and a sharply defined lingual cingulum rimming

the lingual edge of the crown from the mesial end of the preprotocristid to the distal end of the postprotocristid. The $P_4$ of *Nakalipithecus* is larger than in the male of *Anadoluvius* (CO-305), with a less bulging crown surface buccally and a strongly lingually expanded talonid, resulting in considerable crown obliquity. Cingular remnants are also present buccally on the *Nakalipithecus* $P_4$.

*Anadoluvius* is readily distinguished from all other late Miocene hominids. It differs from all dryopithecins (*Dryopithecus, Pierolapithecus, Anoiapithecus, Danuvius, Hispanopithecus* and *Rudapithecus*) in being much larger with more robustly developed facial morphology, large, more thickly enameled molars with broad, rounded cusps, rounded crests, and shallow basins.

*Anadoluvius* differs from *Ankarapithecus* in its shorter, more vertical premaxilla, more pronounced subnasal step, less prognathic mid-face (between the nasal aperture and the malar surfaces), more vertical nasal margins, deeper palate posteriorly, more vertical alveolar process lingually, flatter palatine process lingually (unlike the domed morphology of *Ankarapithecus*), more vertically implanted upper canines, anterolaterally oriented malar surfaces, overlapping superior nasal margin and inferior orbital margins, ethmoidal frontal sinus, thicker interorbital space, lacrimal fossae within orbits, flat superior orbital surfaces (lacking circumorbital rims), more robust upper canines and homomorphic upper premolars.

*Anadoluvius* differs from *Sivapithecus* in many of the same attributes as *Ankarapithecus* and in having a stepped subnasal fossa, biconvex premaxilla, shorter premaxilla, flat facial profile in lateral view, convex profile transversely, broader nasal aperture, anterolaterally oriented zygoma, broader orbits and interorbital space and the absence of circumorbital rims. It differs from *Khoratpithecus* in the same attributes as *Sivapithecus*. It differs from *Indopithecus* in being smaller with a less robust, anteriorly wider mandible and in having more triangular lower premolars and shorter lower molars.

*Anadoluvius* differs from *Lufengpithecus* in its shorter crowned, more vertically implanted canines, broad, low cusped molars and premolars lacking crenulations, more anterolaterally oriented zygoma, absence of circumorbital rims and biconvex frontal trigon.

**Supplementary Note 4: Quantitative analyses**

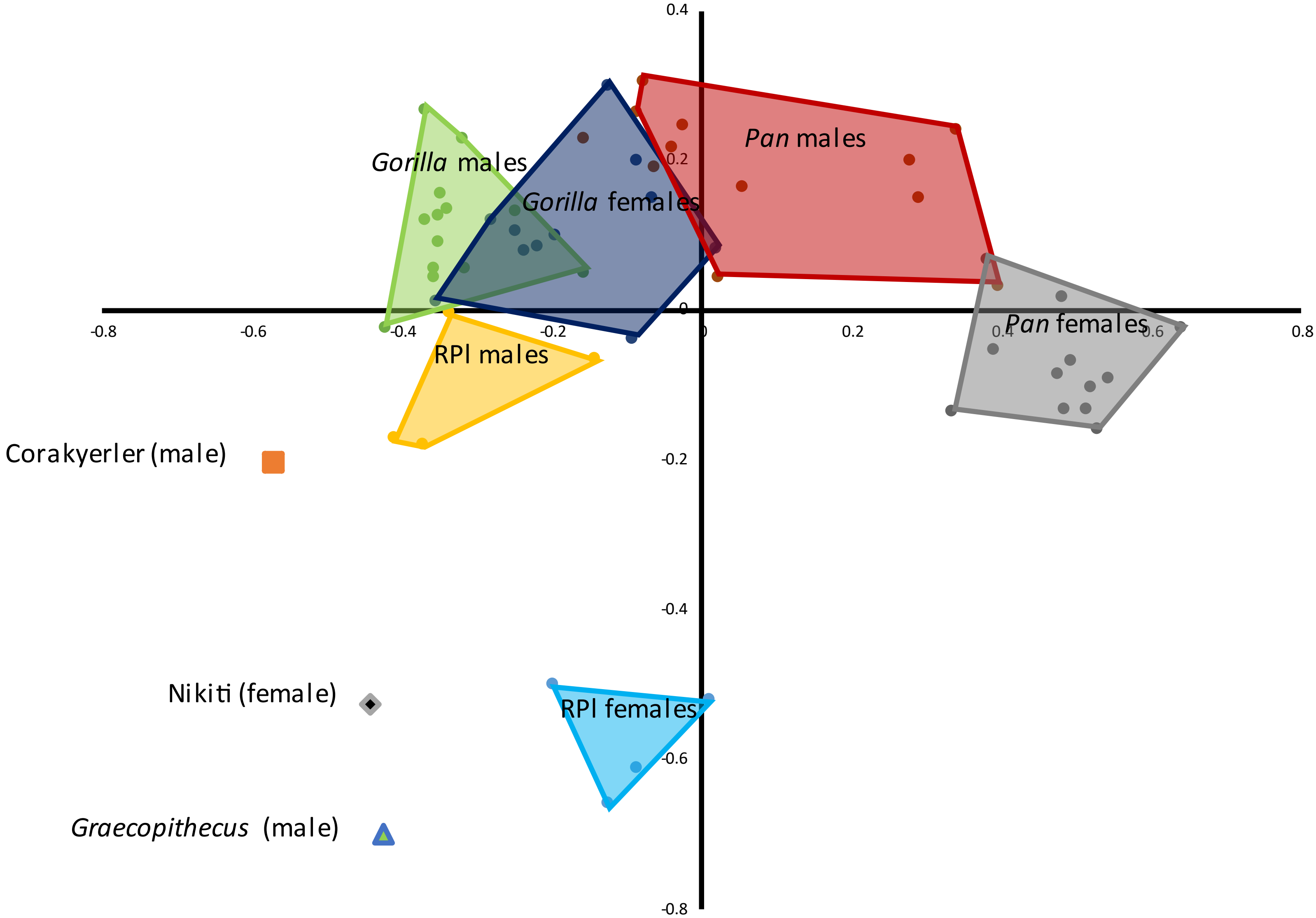


Supplementary Figure 6: PCA from 12 mandibular and dental measurements scaled by the geometric mean (Supplementary Data 1). PC1 and PC2 account for 63 and 24% of the variance respectively. PC1 is driven primarily by mandibular arch breadth (narrower mandibles have more negative values) and PC2 by symphyseal-$M_1$-$M_2$ distance (shorter distances have more negative values; measurements defined in the Methods section). Male and female *Ouranopithecus* are separated from each other and from NKT 21, *Anadoluvius* and G*raecopithecus*. In this and in the subsequent analyses NKT 21 was considered separately as there is a question of its inclusion in *Ouranopithecus macedoniensis* (23). To assess any residual scaling

effects PC1 scores were regressed on the geometric mean, resulting in a low correlation coefficient (0.35), suggesting a small residual size effect.

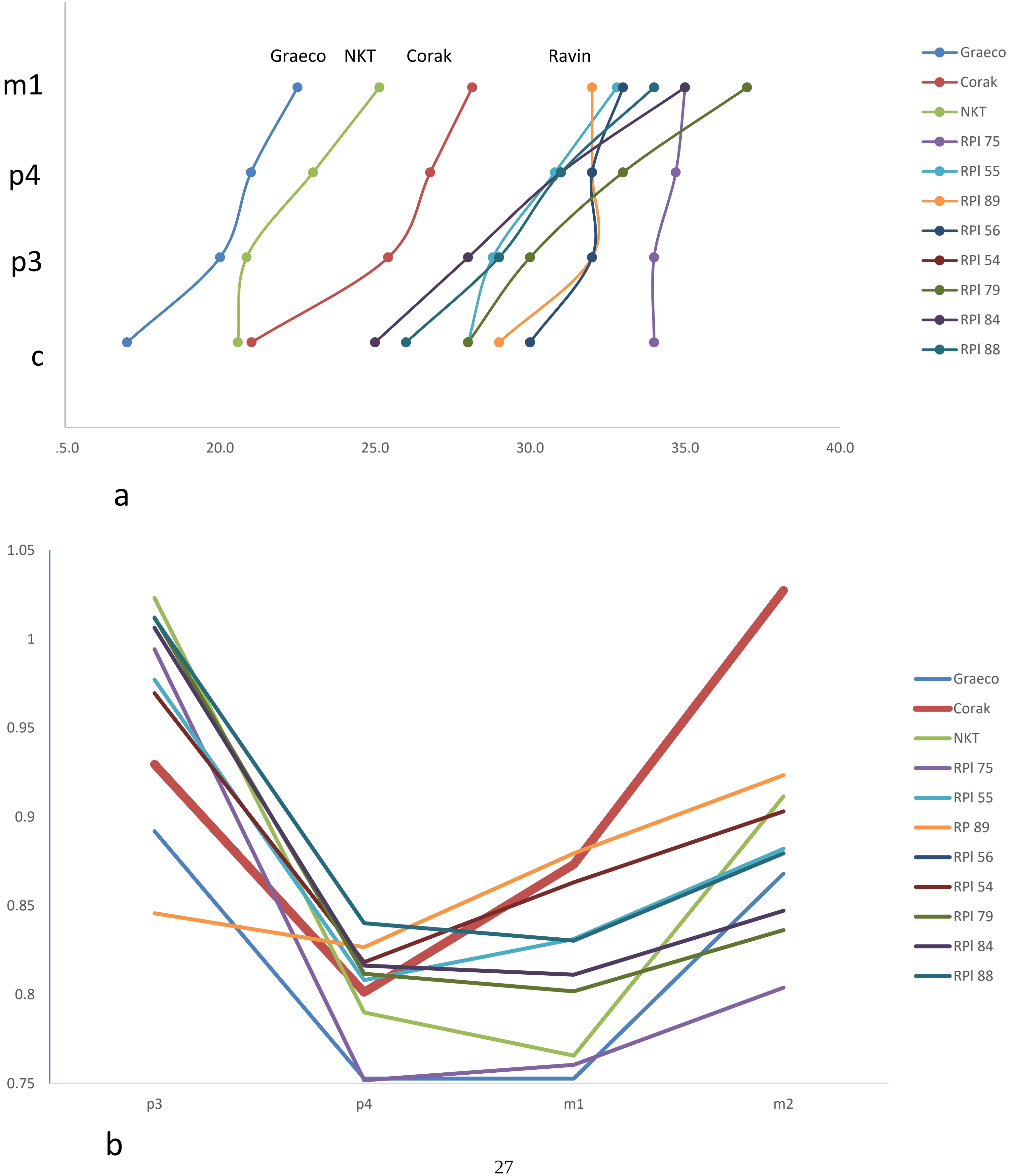
Graeco
NKT
Corak
Ravin
m1
p4
p3
c
.5.0
20.0
25.0
30.0
35.0
40.0
Graeco
Corak
NKT
RPI 75
RPI 55
RPI 89
RPI 56
RPI 54
RPI 79
RPI 84
RPI 88
a
1.05
1
0.95
0.9
0.85
0.8
0.75
p3
p4
m1
m2
Graeco
Corak
NKT
RPI 75
RPI 55
RP 89
RPI 56
RPI 54
RPI 79
RPI 84
RPI 88
b

Supplementary Figure 7. a: Mandibular arch breadth from bi-canine to bi-M1. *Graecopithecus*, NKT 21 and CO-305 have relatively narrow mandibles compared with the combined sex sample of *Ouranopithecus*. This cannot be attributed to size as the Ravin de la Pluie specimens cluster regardless of size and the NKT, *Graecopithecus* and *Anadoluvius* specimens all fall within the ranges for *Ouranopithecus* in dental dimensions. 7b: Mandibular corpus breadth at each postcanine tooth level relative to the geometric mean (thick red line is *Anadoluvius*). The mandibles do not separate clearly at most levels except $M_2$, where CO-305 is noticeably more robust.

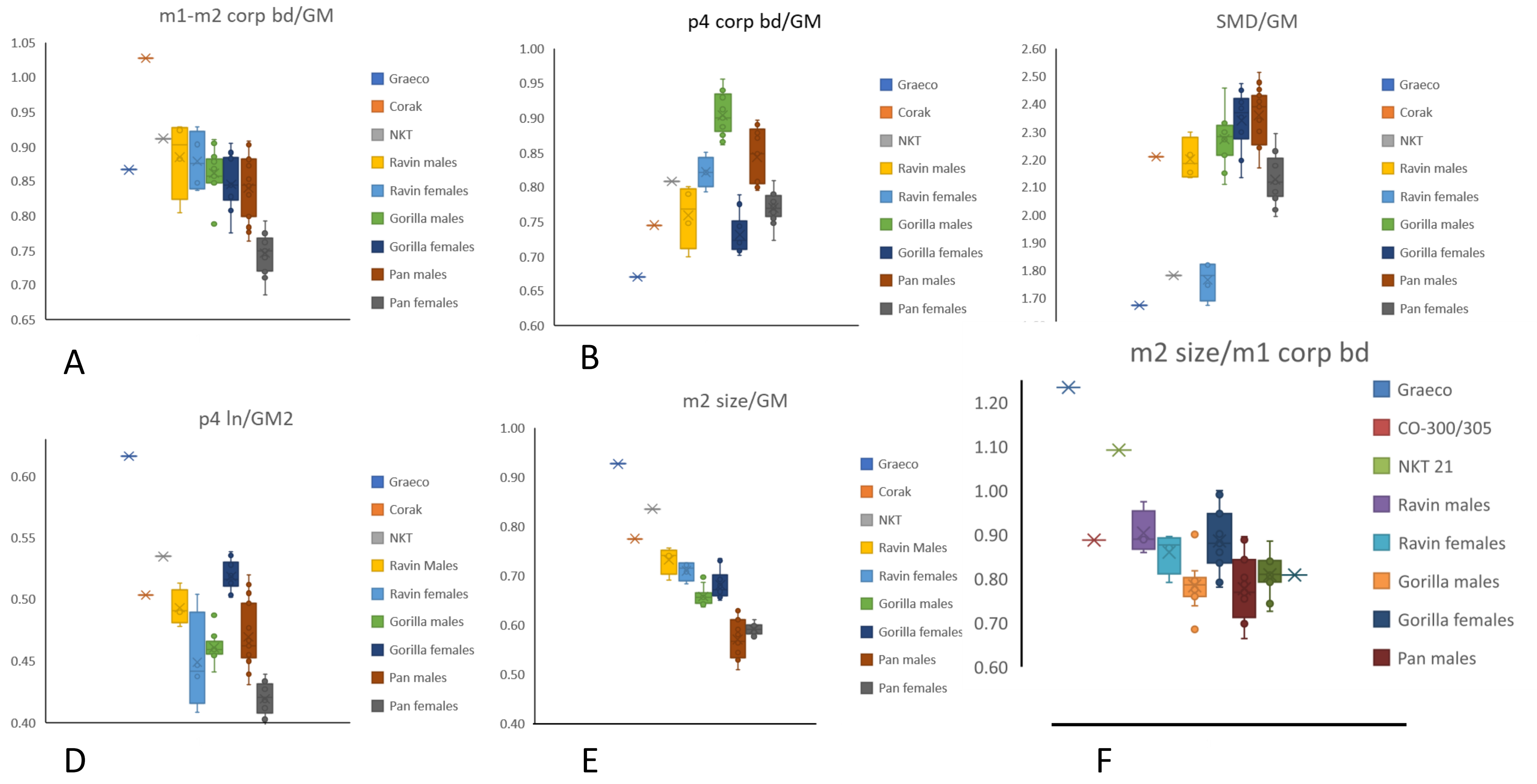


Supplementary Figure 8. 8a-e ratios standardized by the geometric mean. a: Mandibular corpus breadth at m1-m2. *Anadoluvius* has a more robust mandible at this level than any other taxon. 8b: Mandibular corpus breadth at P4. *Anadoluvius* has a more robust mandible at this level than *Graecopithecus*. 8c: Horizontal distance between the symphysis and the corpus between m1 and m2. 8d: P4 relative length. All three non-Ravin de la Pluie samples have relatively elongated lower second premolars. 8e: M2 relative size. All three non-Ravin de la Pluie samples have relatively large lower second molars. *Anadoluvius* is distinct from female *Ouranopithecus*, Nikiti, and

*Graecopithecus*. 8f: $M_2$ size relative to mandibular corpus breadth. This ratio was included to allow comparison with *Nakalipithecus*, which is otherwise too poorly preserved to calculate the same geometric mean used in the other analyses. *Graecopithecus* and NKT 21 both have an enlarged $M_2$ while *Anadoluvius* falls close to the *Ouranopithecus* male mean. *Nakalipithecus* falls outside of the range of variation of *Ouranopithecus* males.

| | CO-300 | *Graecopithecus* | *Ouranopithecus* | *Sahelanthropus* |
|---|---|---|---|---|
| C | $1_1$ (21) | $1_1$ (21) | | |
| $P_3$ | $1_1$M + $1_2$D (21) | $1_1$M+$1_2$D (21) | $1_1$M+$2_2$D (4) | $1_1$M + $1_2$D (2) |
| $P_4$ | $1_1$M + $1_2$D (21) | $1_1$M+$1_2$D (21) | $1_2$M+$2_2$D (2); $2_2$M+$2_2$D (2) | $1_2$M + $1_2$D (2) |
| $M_1$ | $2_2$M + $1_2$D (21) | $2_2$M+$1_{1-2}$D (21) | $2_2$M+$1_2$D (4) | $2_2$M + $1_2$D (2) |
| $M_2$ | | 1-$2_2$M+$1_1$D (21) | $1_2$M+$1_2$D (2); $2_2$M+$1_2$D (3) | $2_2$M + $1_2$D (2) |
| $M_3$ | | $1_1$M+$1_1$D (21) | $1_2$M+$1_1$D (4); $2_2$M+$1_1$D (21) | |
| Ref. | This study | (19) | (19) | (24) |

Supplementary Table 5. Lower tooth root formula in *Anadoluvius*, *Graecopithecus*, *Ouranopithecus* and *Sahelanthropus*. Formula formatted as in (25). Explanation of the formula: $2_2$M + $1_2$D is 2 mesial roots with 2 separate root canals plus 1 distal root with 2 separate root canals. Multiple formulae in *Ouranopithecus* represent variability in that taxon.

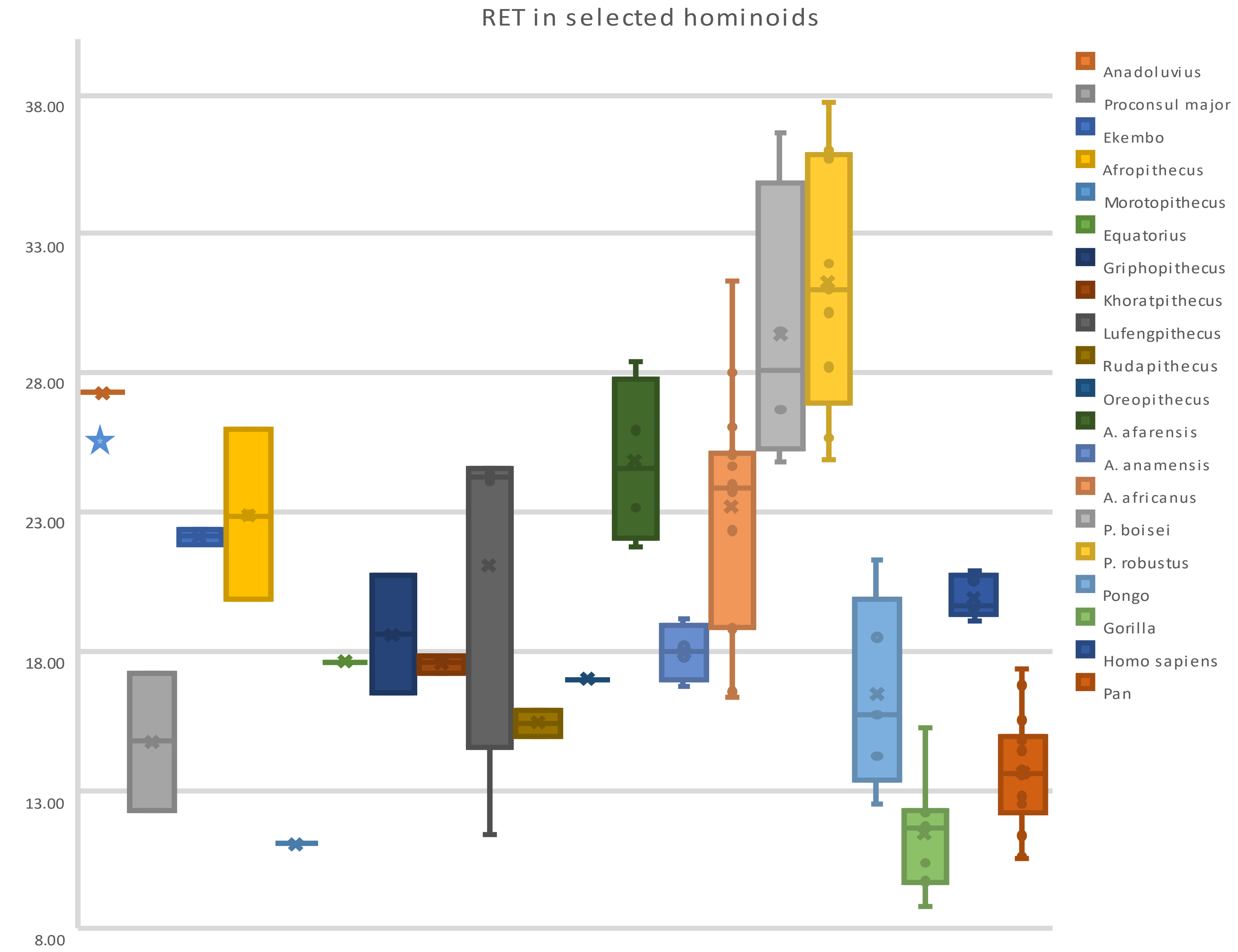


Supplementary Figure 9. Lower M2 relative enamel thickness in selected hominoids. The blue star represents RET in an $M_3$ of *Ouranopithecus* (RET for $M_2$ is not available in *Ouranopithecus*. Data from (26).

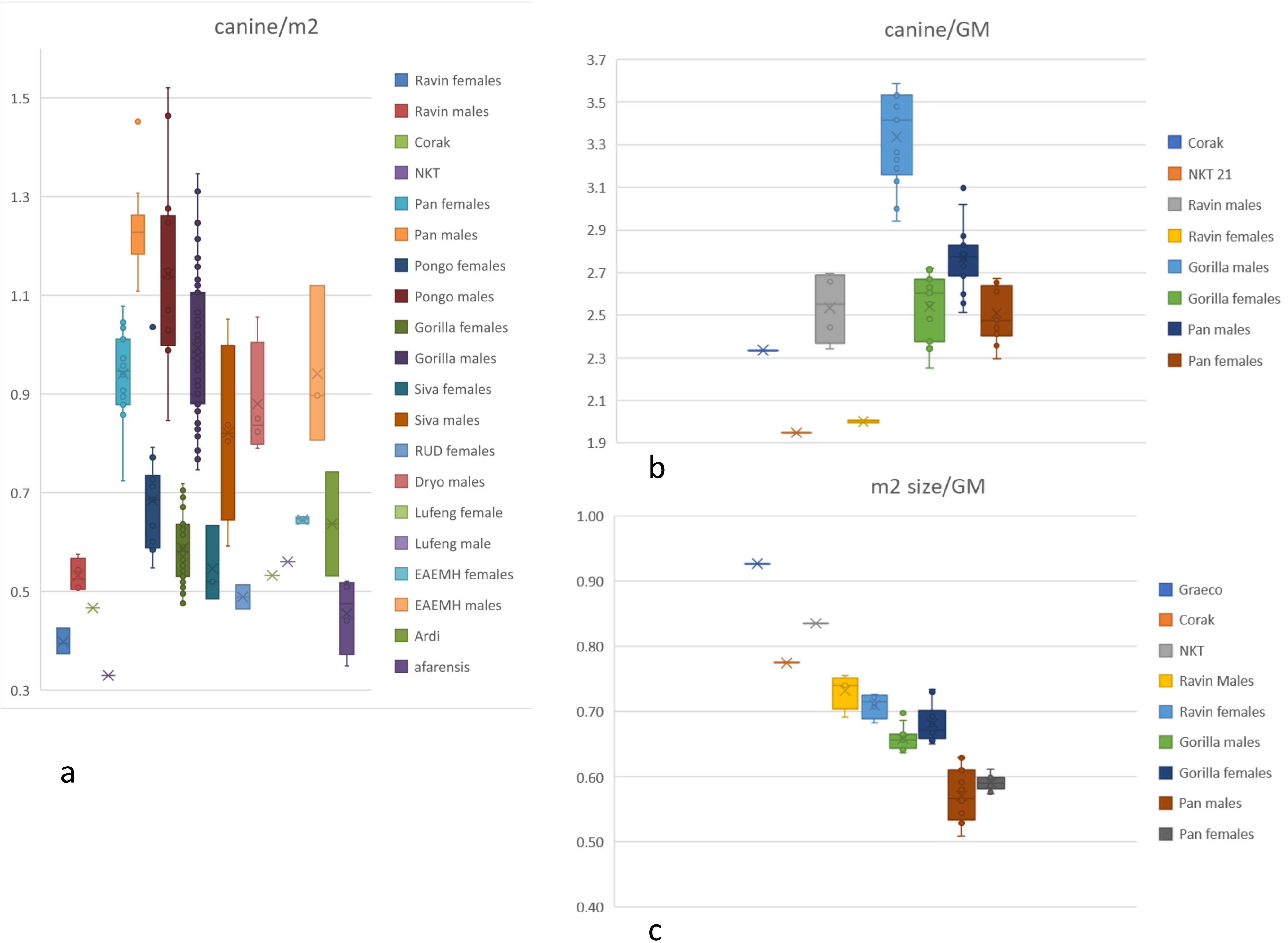
canine/m2
1.5
1.3
1.1
0.9
0.7
0.5
0.3
Ravin females
Ravin males
Corak
NKT
Pan females
Pan males
Pongo females
Pongo males
Gorilla females
Gorilla males
Siva females
Siva males
RUD females
Dryo males
Lufeng female
Lufeng male
EAEMH females
EAEMH males
Ardi
afarensis
a
canine/GM
3.7
3.5
3.3
3.1
2.9
2.7
2.5
2.3
2.1
1.9
Corak
NKT 21
Ravin males
Ravin females
Gorilla males
Gorilla females
Pan males
Pan females
b
m2 size/GM
1.00
0.90
0.80
0.70
0.60
0.50
0.40
Graeco
Corak
NKT
Ravin Males
Ravin females
Gorilla males
Gorilla females
Pan males
Pan females
c

Supplementary Figure 10. a: Canine relative to m2. EAEMH= East African early Miocene hominoids. Eastern Mediterranean apes fall within the ranges of hominins and *Lufengpithecus*. Canine and lower m2 size (ln x bd) comparisons. All size values were scaled to the same power (e.g. square root of ln x bd when divided by the geometric mean. 10b: Canine size relative to the geometric mean confirms the small size of the canines in eastern Mediterranean apes compared with extant African apes. Using the GM avoids, at least in part, the issue of co-variation in the simple size ratio of canine and m2, which is more common in assessments of relative canine size. 10c: Lower m2 size (lnxbd) relative to the geometric mean confirms the relatively large size of the molars in eastern Mediterranean apes.

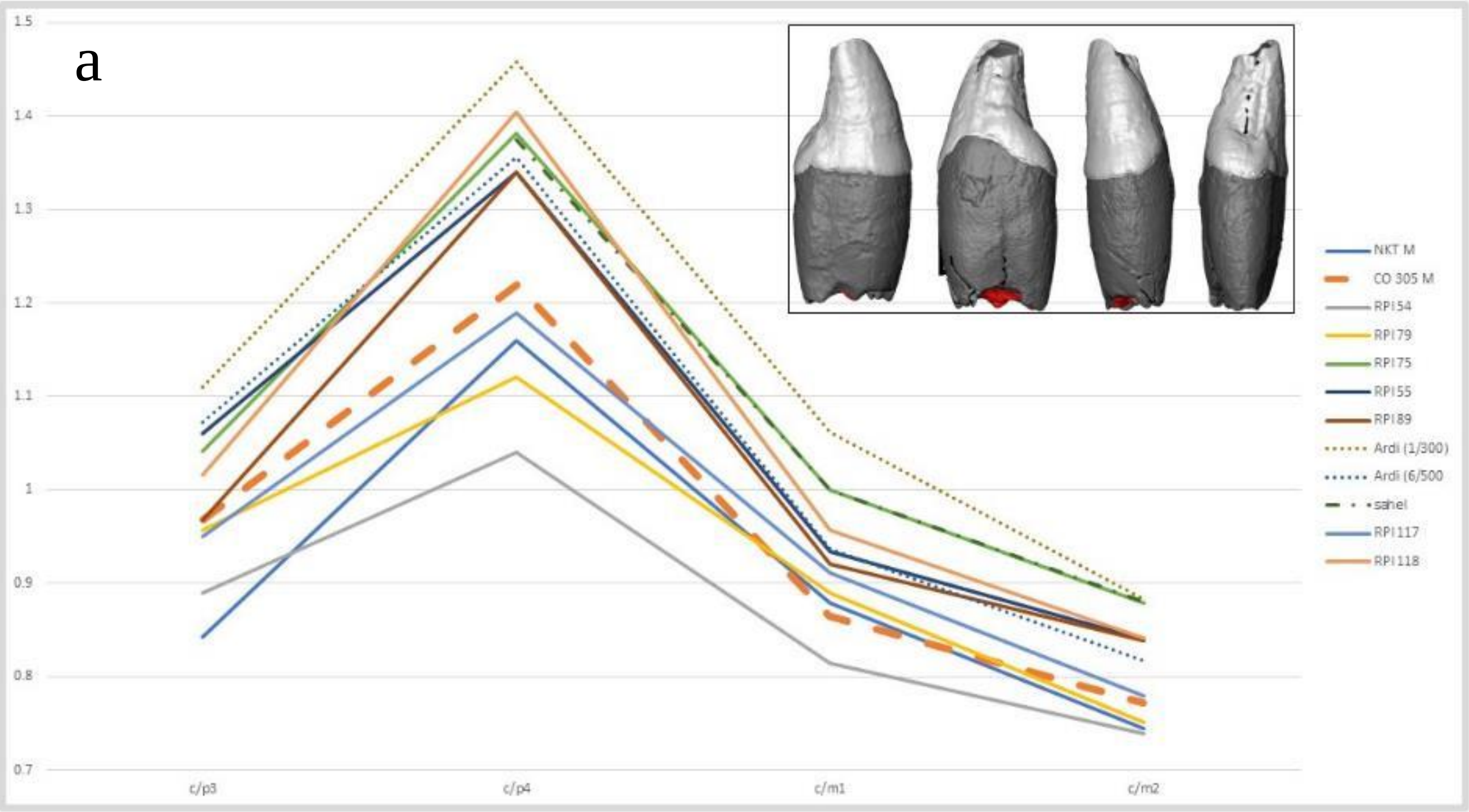
a
1.5
1.4
1.3
1.2
1.1
1
0.9
0.8
0.7
c/p3
c/p4
c/m1
c/m2
NKT M
CO 305 M
RPI54
RPI79
RPI75
RPI55
RPI89
Ardi (1/300)
Ardi (6/500
sahel
RPI117
RPI118

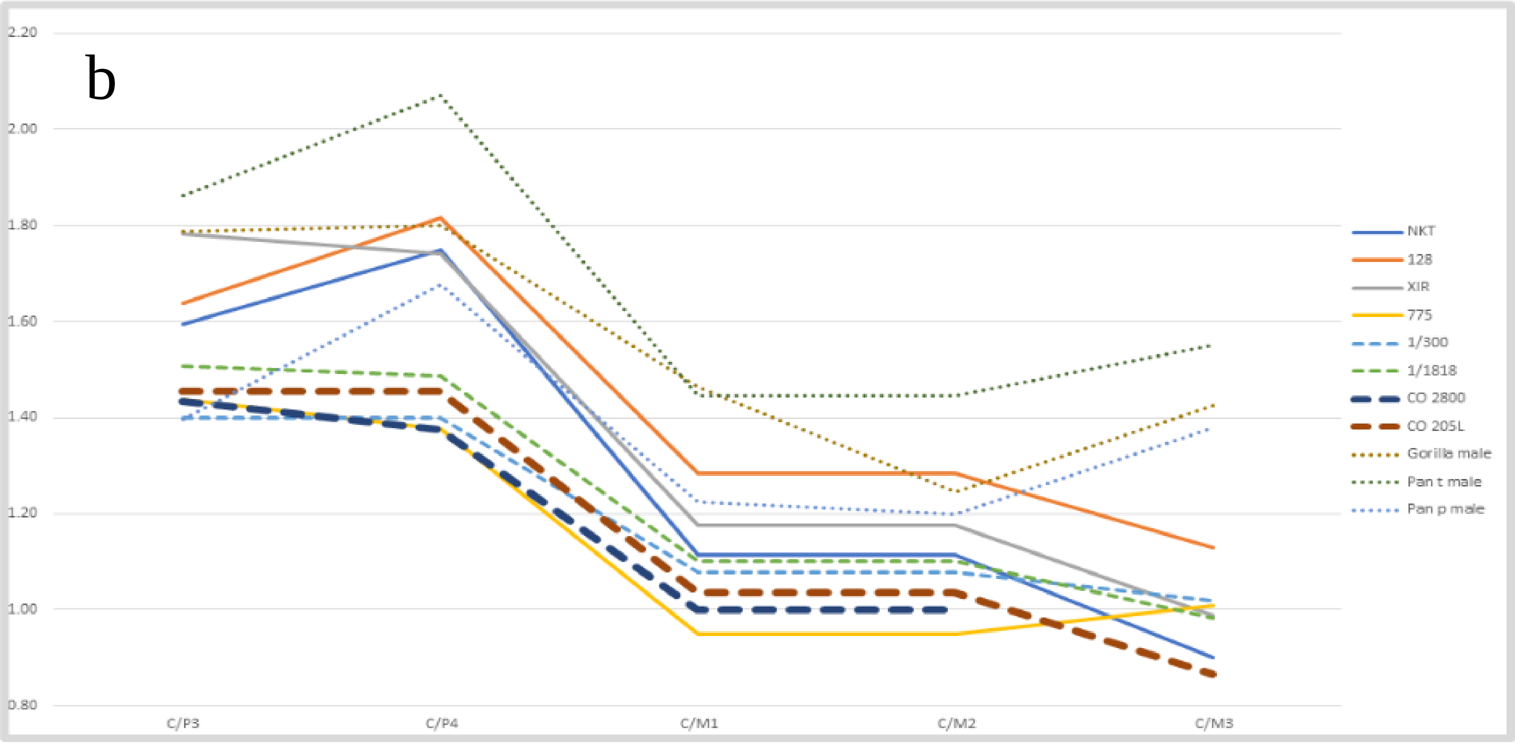
b
2.20
2.00
1.80
1.60
1.40
1.20
1.00
0.80
C/P3
C/P4
C/M1
C/M2
C/M3
NKT
128
XIR
775
1/300
1/1818
CO 2800
CO 205L
Gorilla male
Pan t male
Pan p male

Supplementary figure 11. a: Lower canine size relative to each lower postcanine tooth. The inset shows the digitally extracted canine of CO-305 in lingual, buccal, mesial and distal views. The relative size of the lower canine in the male CO-305 (thick red dashed line) falls among female *Ouranopithecus* while male *Ouranopithecus* groups with *Ardipithecus* and *Sahelanthropus.* The association of the African hominins with male *Ouranopithecus* probably reflects the decreased size of the postcanine dentition in these taxa (see discussion). The pattern in the upper canines differs, with the two *Anadoluvius* specimens clustering with the single *Ouranopithecus* female (RPl 775) and *Ardipithecus* while male *Ouranopithecus* falls among male *Pan* and *Gorilla*. Data on *Sahelanthropus*, *Ardipithecus* and *Au. afarensis* from (15,16,27). 11 b: Upper canine size relative to each upper postcanine tooth. The pattern in the upper canines differs from the lower canines (Supplementary Figure 11a). The two *Anadoluvius* specimens cluster with the single *Ouranopithecus* female (RPl 775) and *Ardipithecus* while male *Ouranopithecus* falls among male *Pan* and *Gorilla*. Data on *Sahelanthropus*, *Ardipithecus* and *Au. afarensis* from (15,16,27).

| Statistic | *Ouranopithecus* ♂ | *Ouranopithecus* ♀ | *Gorilla* ♂ | *Gorilla* ♀ | *Pan* ♂ | *Pan* ♀ |
|---|---|---|---|---|---|---|
| N | 4 | 3 | 13 | 11 | 15 | 14 |
| Min | 0.49 | 0.44 | 0.58 | 0.49 | 0.53 | 0.51 |
| Max | 0.56 | 0.45 | 0.74 | 0.58 | 0.72 | 0.66 |
| Mean | 0.53 | 0.44 | 0.66 | 0.54 | 0.61 | 0.61 |
| Standard deviation | 0.03 | 0.002 | 0.06 | 0.03 | 0.05 | 0.05 |

Supplementary Table 6: Summary statistics for canine size (square root of maximum length X perpendicular breadth divided by the geometric mean of 12 mandiblular and dental measurements (see Supplementary Data 1).

| | Ourano male | Ourano female | Gorilla male | Gorilla female | Pan male | Pan female |
|---|---|---|---|---|---|---|
| Ourano male | | 0.1164 | 0.00012 | 1 | 0.03921 | 0.06454 |
| Ourano female | 3.668 | | 9.995E-09 | 0.03012 | 4.363E-06 | 8.796E-06 |
| Gorilla male | 6.957 | 10.58 | | 1.93E-07 | 0.05825 | 0.03387 |
| Gorilla female | 0.1739 | 4.457 | 9.462 | | 0.001616 | 0.004283 |
| Pan male | 4.314 | 8.298 | 4.091 | 5.86 | | 0.9998 |
| Pan female | 4.031 | 7.996 | 4.394 | 5.42 | 0.3828 | |

Supplementary Table 7: One-way ANOVA (Tukey's pairwise) on canine size relative to GM (p above the diagonal, Q below.) Shaded cells are significantly different. Samples: *Ouranopithecus* (4♂, 3♀); *Gorilla* (13♂, 11♀); *Pan* (15♂, 14♀). *Ouranopithecus* males are not significantly different from *Ouranopithecus* females and African ape females. *Ouranopithecus* males are significantly different in relative canine size from male African apes, which are significantly different from African ape females. *Ouranopithecus* females differ from all other groups

**Supplementary Note 5: Phylogenetic Analysis**

Characters and character states used in this analysis[1]. Characters that were ordered are noted.

1. Frontal squama inclination: 0=vertical; 1=inclined. Angle of the frontal squama in lateral view.

2. Frontal trigone: 0=biconvex, 1=flat, 2=biconcave, 3=convex. The region between the temporal lines and the supraorbital region.

3. Postorbital constriction: 0=deep, 1=moderate, 2=wide. Maximum constriction relative to bi-orbital breadth. **Ordered**

4. Supraorbital margin: 0=sharp, 1=rounded, 2=rimmed. Most anterior edge of the orbital margin at the junction with the frontal squama.

5. Supraorbital notch: 0=shallow, 1=deep, 2=absent

6. Supraorbital torus: 0=absent, 1=present. Defined as an elongated, palpable thickened area arising bilaterally from glabella and running superior to the supraorbital margin.

7. Supratoral sulcus: 0=absent, 1=pronounced, 2=moderate. Variably deep sulcus on the frontal squama posterior to the supraorbital torus. **Ordered**

8. Frontal sinus/glabella: 0=above, 1=below, 2=absent. The position of the frontal sinus relative to glabella.

9. Frontal-ethmoidal sinus: 0=separate, 1=connected. Continuity or lack thereof of a frontal sinus, positioned at least as far anteriorly as glabella and the ethmoid sinus, identified as the paranasal sinus positioned below nasion along the medial orbital margin.

10. Interorbital space: 0=broad, 1=intermediate, 2=narrow. Assessed relative to orbital dimensions. **Ordered**

11. Orbital shape: 0=broad, 1=tall. Maximum height of the orbital relative to maximum breadth.

12. Lateral orbital pillar: 0=narrow, 1=broad. Assessed relative to orbital dimensions.

13. Lateral orbital pillar surface orientation: 0=lateral, 1=anterior. The orientation of the surface consisting of the frontal process of the zygomatic and the maxillary process of the frontal between the lateral orbital margin and the lateral edge of the orbital region.

14. Lateral orbital margin: 0=sharp, 1=rounded. Junction between the lateral orbital surface and the lateral margin of the orbit.

15. Inferior orbital margin: 0=sharp, 1=rounded. Junction between the maxillary and zygomatic orbital processes and the inferior edge of the orbit.

16. Nasomaxillary surface orientation: 0=lateral, 1=anterior. Orientation of surface of the maxilla immediately lateral to the margin of the nasal aperture.

17. Maxillary frontal process shape: 0=flat, 1=concave. Shape of the surface of the maxillary frontal process adjacent to the inferomedial corner of the orbit.

18. Naso-orbital breadth: 0=wide, 1=narrow. Shortest distance between the orbital and nasal margins assessed relative to overall facial size.

19. Nasal bone length: 0=long, 1=strongly elongated, 2=short. Assessed relative to upper facial dimensions.

20. Nasal aperture breadth: 0=intermediate, 1=broad, 2=narrow. Maximum breadth of the nasal aperture superior to the base in comparison with overall midfacial size.

21. Nasal base shape: 0=notched, 1=flat. The shape of the surface of the nasoalveolar process that forms the inferior margin of the nasal aperture.

22. Nasal base breath: 0=narrow, 1=intermediate, 2=broad. Assessed relative to overall midfacial dimensions. **Ordered**

23. Frontonasal suture angle: 0=obtuse, 1=acute, 2=rounded

24. Nasal bone breath: 0=broad, 1=narrow. Assessed relative to overall interorbital size.

25. Nasion height: 0=below orbit, 1=at orbit. Position of nasion relative to a line connecting the most superior edges of the superior orbital margins in frontal view.

26. Premaxillary nasal surface: 0=anterolateral, 1=anterior. Orientation of the surface of the premaxilla adjacent to the inferolateral corner of the nasal aperture.

27. Interorbital sagittal curvature: 0=flat, 1=concave. Shape of the plane of the interorbital surface in lateral view.

28. Inferior nasal margins: 0=sharp, 1=inflated. Shape of the inferolateral margin of the nasal aperture above and below the inferolateral corner.

29. Nasal inclination: 0=inclined, 1=vertical. Inclination of the nasal bones in lateral view.

30. Nasal projection: 0=strong, 1=weak. Projection of the inferior margins of the nasal bones relative to the adjacent maxillary surface in lateral view.

31. Nasal apex/orbit: 0=narrow, 1=wide. Distance between the most superior point of the nasal aperture and a line connecting the most inferior margins of the orbits.

32. Zygomatic body: 0=lateral, 1=anterior. Orientation of the surface of the zygomatic bone inferior to the inferior orbital margin.

33. Zygomatic root: 0=low, 1=high. Position of the zygomaticoalveolar crest relative to the lateral edge of the maxillary alveolar process.

34. Zygomaticoalveolar crest: 0=sharp, 1=broad. Shape of the zygomaticoalveolar crest immediately superior to its root on the maxilla.

35. Canine orientation: 0=inclined, 1=vertical. Orientation of the long axis of the canine in lateral view.

36. Subnasal floor: 0=flat, 1=stepped. Position of the base of the nasal aperture relative to the palatine process of the maxilla.

37. Clivus length: 0=very short, 1=short, 2=intermediate, 3=long. Distance from prosthion to the intranasal margin of the premaxilla. **Ordered**

38. Clivus orientation: 0=vertical, 1=inclined, 2=strongly inclined. Inclination of the nasoalveolar clivus in lateral view. **Ordered**

39. Clivus shape: 0=biconvex, 1=flat, 2=convex. Shape of the surface of the nasoalveolar clivus in the transverse plane.

40. Palatal fenestration: 0=large, 1=absent. The midline gap between the palatine processes of the premaxilla and maxilla.

41. Incisive canal: 0=absent, 1=short, 2=long, 3=very long. Canal formed by the overlap of the nasoalveolar process and the anterior most portion of the palatal maxilla, assessed in comparison with the overall lower facial dimensions. **Ordered**
42. Incisive foramen position: 0=canine, 1=P3, 2=canine-P3. Position of the posterior edge of the incisive foramen relative to the dentition. **Ordered**
43. Incisive foramen size: 0=absent, 1= very small, 2=small, 3= large. Foramen coded as absent in the presence of incisive fenestration. Size assessed relative to anterior palatal breadth. **Ordered**
44. Diastema (I2-Canine): 0=small, 1=absent, 2=large. Assessed in comparison with the anterior dental size.
45. Palatal length: 0=short, 1=elongated. Assessed in comparison with overall palatal dimensions.
46. Anterior palatal depth: 0=shallow, 1=deep. Depth of the palate relative to the alveolar process in the region of the canines and premolars.
47. Posterior palatal breadth: 0=broad, 1=narrow. Distance between the lingual surfaces of the molar alveolar processes in comparison with overall palatal dimensions.
48. Posterior palatal depth: 0=shallow, 1=deep, 2=very deep. Depth of the palate relative to the alveolar process in the region of the molars. **Ordered**
49. I1 length: 0=intermediate, 1=long, 2=short. Mesiodistal dimension in comparison with crown breadth and height.
50. I1 breadth: 0=short, 1=long. Labiolingual dimension in comparison with crown length and height.

51. I1 cingulum: 0=sharp, 1=absent, 2=mild. Definition of the lingual cingulum continuous with the mesial and distal marginal ridges. **Ordered**

52. I1 lingual bulge: 0=absent, 1=broad, 2=narrow. Lingual crown prominence between the cingulum and the cervix.

53. I1 median pillar: 0=absent, 1=present. Centralized ridge of variable thickness on the labial surface of the crown between the cingulum and incisive edge.

54. I1 median pillar shape: 0=absent, 1=thick, 2=narrow.

55. I1 marginal ridge shape: 0=sharp, 1=thick, 2=moderate. Ridges of variable development along the mesial and distal margins of the crown.

56. I1 marginal ridge symmetry: 0=symmetric, 1=asymmetric.

57. I2: 0=peg-shaped, 1=diamond-shaped, 2=spatulate. As defined by overall size, the angle formed between the mesial and distal marginal ridges, and whether the edges meet in a point at the crown apex.

58. I2 root/nasal aperture: 0=in-line, 1=medial, 2=lateral. Position of the root of the upper lateral incisor relative to the margins of the nasal aperture.

59. I2 sagittal orientation: 0=inclined, 1=vertical. Inclination of the upper lateral incisor crown and root in lateral view.

60. I2 lingual cingulum: 0=sharp, 1=thick, 2=absent. Same as for I1.

61. I2 mesial marginal ridge shape: 0=sharp, 1=thick. Same as for I1

62. I2 distal marginal ridge shape: 0=angled, 1=vertical. Orientation of the distal marginal ridge relative to the mesial marginal ridge and the long axis of the tooth.

63. C/M1 bd: 0=subequal, 1=smaller. Upper canine breadth relative to upper molar breadth.

64. C crown shape: 0=thick, 1=narrow. Upper canine crown maximum breadth relative to maximum length.

65. C shoulders: 0=short, 1=intermediate, 2=tall. Height of the crown from the cervix to the cingulum. **Ordered**

66. C lingual tubercle: 0=absent, 1=present. Accessory cusp arising from the protocone.

67. Canine lingual cingulum: 0=present, 1=absent. Cingulum of variable development between the mesial and distal margins of the crown.

68. Canine mesial groove: 0=deep, 1=shallow.

69. Canine implantation: 0=medial, 1=vertical. Inclination of the canine crown and root in frontal view.

70. Canine inclination: 0=posterior, 1=subvertical, 2=strongly posterior. Inclination of the canine crown and root in lateral view.

71. Canine lingual bulge: 0=absent, 1=mild, 2=pronounced. Development of the crown between the mesial and distal shoulders, between the cingulum and cervix. **Ordered**

72. P3 shape: 0=triangular, 1=rectangular. In occlusal view.

73. P3 mesial margin shape: 0=concave, 1=flat, 2=convex. In occlusal view. **Ordered**

74. P3 mesial marginal ridge shape: 0=sharp, 1=rounded. Mesial ridge between the mesiolingual corner of the crown and the paracone apex.

75. P3 cusps: 0=long, 1=equal, 2=short. Compared with overall crown dimensions. **Ordered**

76. P3 protocone: 0=tall, 1=short. Compared with the paracone of the same tooth.

77. P3-P4: 0=heteromorphic, 1=homomorphic. Degree to which the P4 resembles the P3 in occlusal outline and cusp development.

78. P4 cusps: 0=conical, 1=broad, 2=rounded. Shape of the cusp from the base to the apex.

79. Upper premolar cusps: 0=separate, 1=joined. Bases in contact or cusps spaced apart.

80. Upper premolar crests: 0=pronounced, 1=absent, 2=interrupted. Crests arising from the protocone and paracone continuous or divided by a cleft.

81. Upper premolar length: 0=short, 1=long. In comparison with breadth and overall tooth dimensions.

82. Upper premolar lingual surface: 0=flared, 1=vertical. Orientation of the crown surface lingual to the occlusal margin.

83. Upper molars: 0=broad, 1=long. Compared with overall crown dimensions.

84. Upper molar flare: 0=pronounced, 1=weak. As for upper premolar.

85. Upper molar lingual cingulum: 0=pronounced, 1=absent.

86. Upper molar protocone shape: 0=compressed, 1=expanded. Surface area of the upper molar protocone relative to the other cusps and to overall crown dimensions.

87. canine shape: 0=broad, 1=compressed. Lower canine breadth compared with crown length and overall dimensions.

88. canine mesial groove: 0=deep, 1=shallow, 2=absent. Lower canine. **Ordered**

89. canine distal groove: 0=absent, 1=present. Lower canine.

90. canine lingual cingulum: 0=pronounced, 1=weak.

91. cingulum orientation: 0=horizontal, 1=oblique. Inclination of the lower canine cingulum relative to the mesial and distal margins.

92. canine mesiobuccal crown flare: 0=absent, 1=weak, 2=moderate, 3=pronounced. Mesiobuccal expansion of the lower canine crown relative to the cervix. **Ordered**

93. canine height: 0=tall, 1=short. In comparison with overall canine dimensions.

94. p3 mesiobuccal flare: 0=strong, 1=weak, 2=intermediate. Development of the mesiobuccal crown surface associated with honing of the upper canine's distal margin. **Ordered**

95. p3 mesiobuccal surface: 0=flat, 1=bulging. As for lower canine.

96. p3 protoconid height: 0=tall, 1=short. In comparison with the metaconid and talonid.

97. p3 metaconid: 0=absent, 1=present.

98. p3 distal marginal ridge: 0=short, 1=long. Distal border of the distal fovea.

99. p3 shoulders: 0=short, 1=tall. Height of the crown between the cervix and cingulum.

100. p3 lingual/distal ridge ratio: 0=long, 1=short. Length of the lingual marginal ridge from the mesial beak/mesiolingual corner to the distolingual corner compared with the length of the distal marginal ridge, between the buccal and lingual margins.

101. p3 distolingual corner: 0=sharp, 1=rounded. Junction between the lingual and distal margins.

102. p3 hypoprotocristid-postprotocristid angle: 0=low, 1=high. Angle between the transversely oriented hypoprotocristid and the distally oriented postprotocristid.

103. p4 shape: 0=short, 1=long. In comparison with overall tooth dimensions p3 shape.

104. p4 trigonid height: 0=tall, 1=low. Degree of elevation of the mesial moiety of the crown (protoconid-metaconid) relative to the talonid.

105. Lower molars: 0=broad, 1=long. In comparison with overall molar dimensions

106. Lower molar cingula: 0=prominent, 1=slight, 2=absent. Shelf-like, ridge-like or missing. **Ordered**

107. Lower molar cusp relations: 0=isolated, 1=connected. Position of the cusp bases and separation of the cusp apices.

108. Lower molar cusps: 0=conical, 1=broad. From base to cusp tip.

109. Lower molars lingual flare: 0=strong, 1=intermediate, 2=weak. Orientation of the lingual crown surface below the occlusal margin. **Ordered**

110. Buccal cristids: 0=notched, 1=continuous. Connected or indented postprotocristid and prehypocristid.

111. m2 hypoconulid: 0=short, 1=intermediate, 2=long. In comparison with the other cusps and overall tooth dimensions. **Ordered**

112. Enamel thickness: 0=thick, 1=hyperthick, 2=thin, 3=intermediate. As measured and classified in the literature.

Notes: 1. Attributes (e.g. nasal bone length) were not quantified but coded as discrete characters assessed relative to adjacent cranial structures. Discrete coding of quantitative characters has been shown to introduce reduced reliability in cladistic analyses in a similar sample of Miocene apes (28). In that study, the author finds the use of discretized characters problematic and favors continuous quantitative characters scaled by first molar dimensions. However, simple ratios fail to account for allometric effects. The use of first molar dimensions is also problematic given observed differences in relative dental size (microdonty vs megadonty) among Miocene apes.

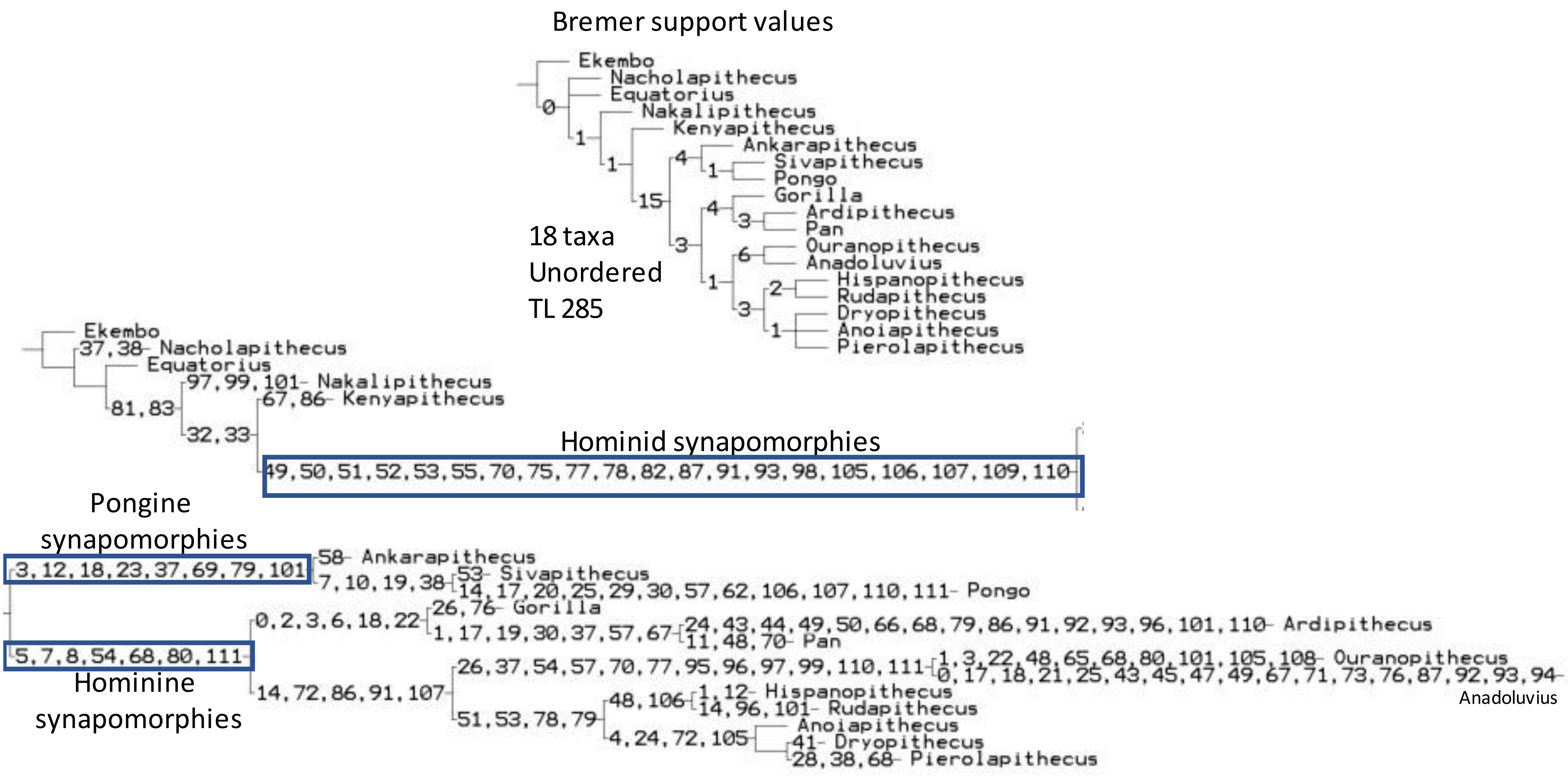


Supplementary Figure 12: Bremer support values and synapomorphies mapped onto the consensus cladogram from the unordered analysis of 18 taxa.

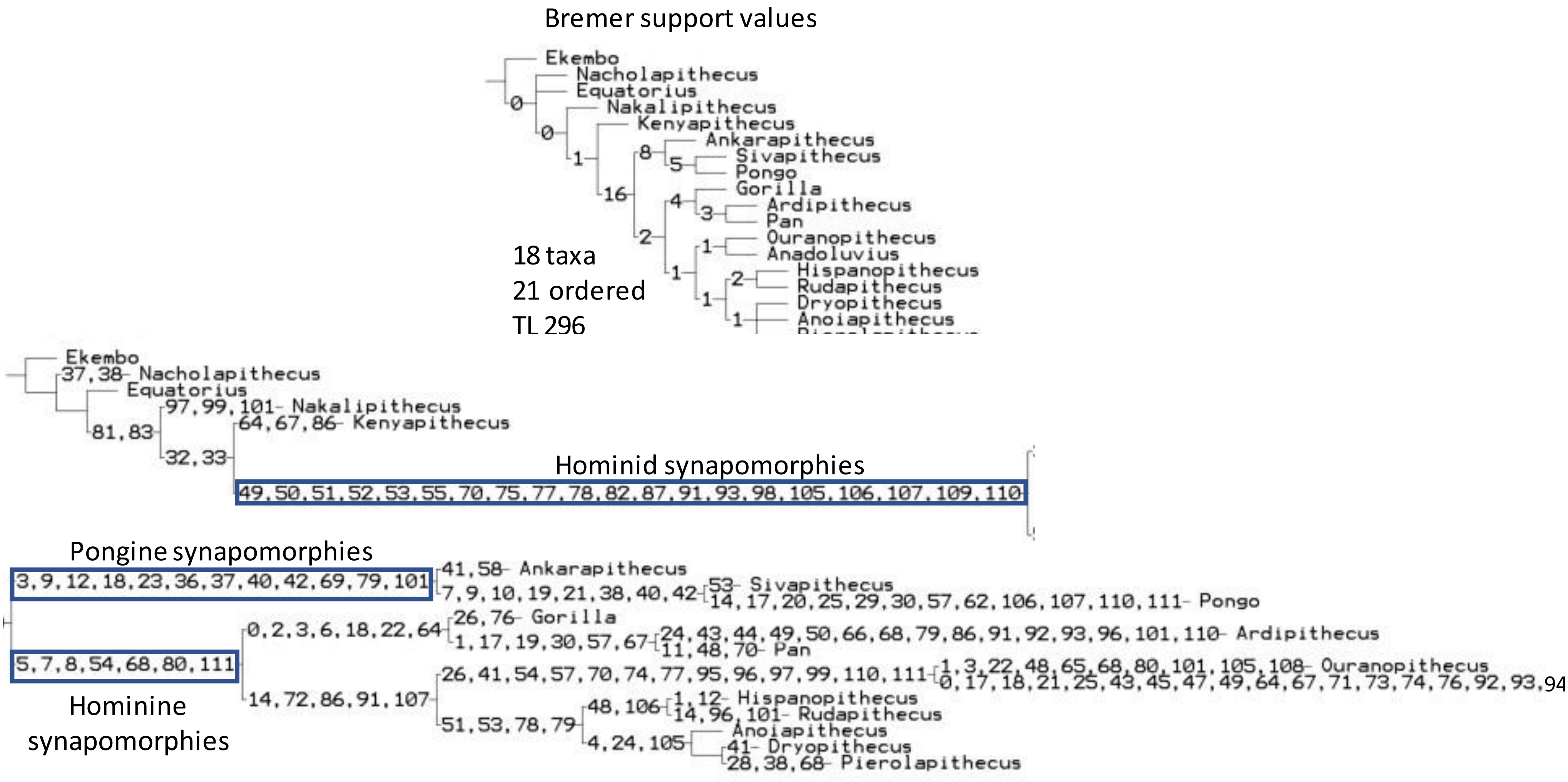


Supplementary Figure 13: Bremer support values and synapomorphies mapped onto the consensus cladogram from the partly ordered analysis of 18 taxa.

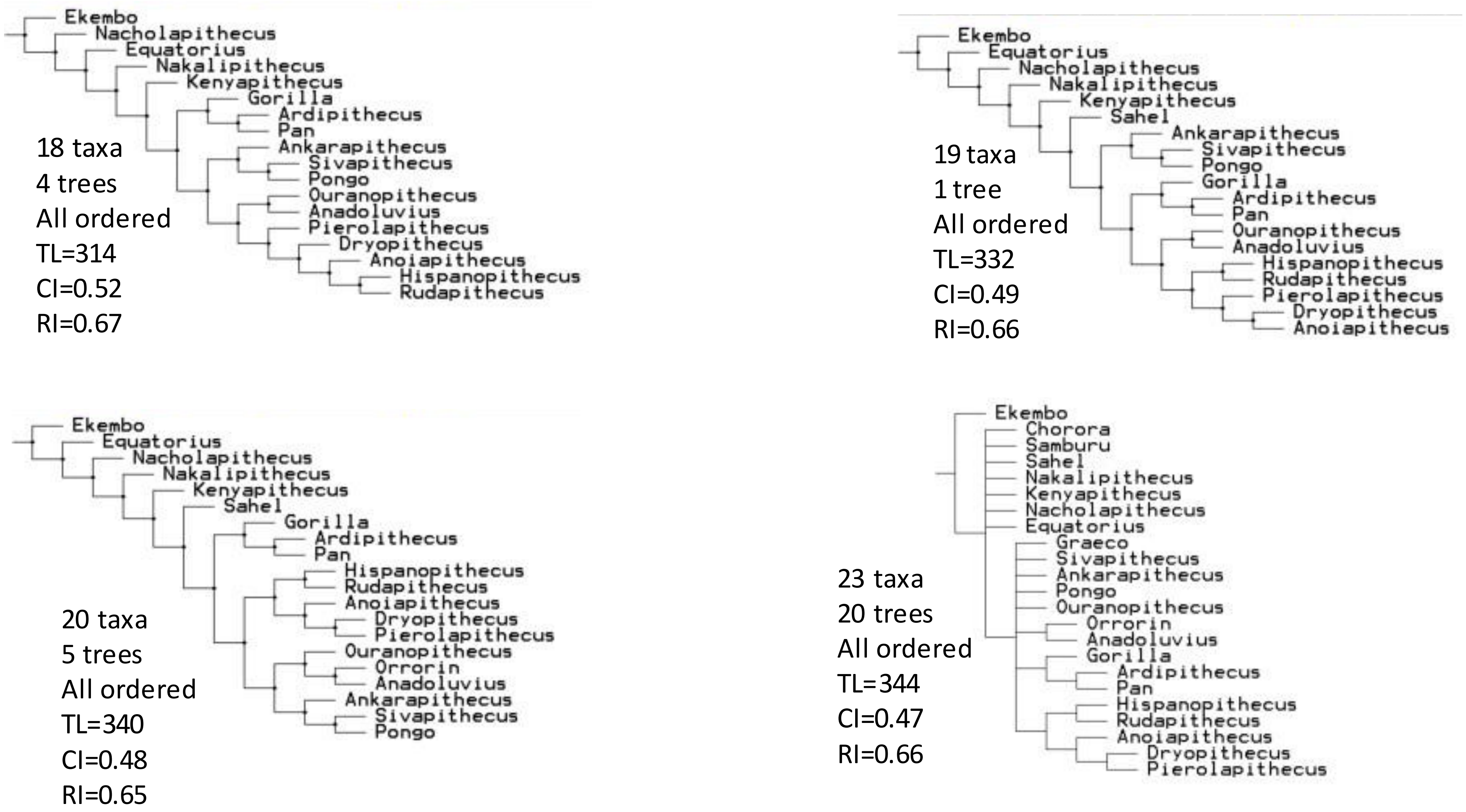


Supplementary Figure 14: Consensus cladograms for 18, 19, 20 and 23 taxa with all 112 characters ordered. See text for discussion.

**Supplementary Note 6: Historical, geological and taxonomic background**

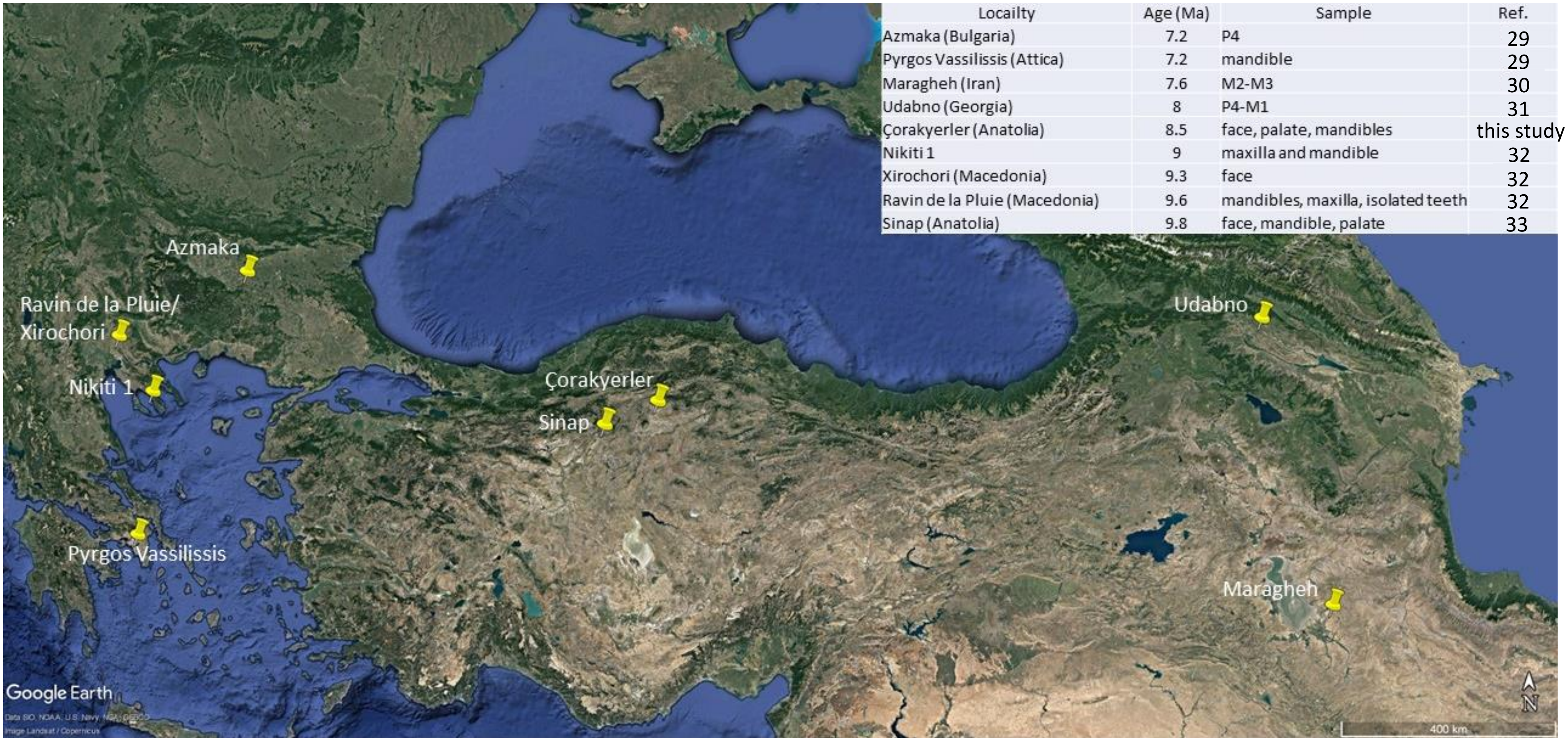


| Locailty | Age (Ma) | Sample | Ref. |
|---|---|---|---|
| Azmaka (Bulgaria) | 7.2 | P4 | 29 |
| Pyrgos Vassilissis (Attica) | 7.2 | mandible | 29 |
| Maragheh (Iran) | 7.6 | M2-M3 | 30 |
| Udabno (Georgia) | 8 | P4-M1 | 31 |
| Çorakyerler (Anatolia) | 8.5 | face, palate, mandibles | this study |
| Nikiti 1 | 9 | maxilla and mandible | 32 |
| Xirochori (Macedonia) | 9.3 | face | 32 |
| Ravin de la Pluie (Macedonia) | 9.6 | mandibles, maxilla, isolated teeth | 32 |
| Sinap (Anatolia) | 9.8 | face, mandible, palate | 33 |

Supplementary Figure 15. Satellite image of the eastern Mediterranean and the Anatolian peninsula with hominid localities.

Inset: List of hominid localities, their ages, and a summary of the known fossils.

*Taxonomic background*

Large apes have been known from late Miocene deposits in the Balkans since 1951 (34). Most fossils come from Macedonia (northern Greece), with scattered specimens from Attica (southern Greece) and Bulgaria (Supplementary Figure 15). These fossils are usually assigned to *Ouranopithecus* or *Graecopithecus* (10). In Anatolia, late Miocene *Ankarapithecus* is probably a pongine (35, but see 11,36), while a small sample from Çorakyerler has been attributed to *Ouranopithecus* (21).

Late Miocene Balkan apes were first described from Pyrgos Vassilissis in Athens, based on a single battered mandible attributed to the genus *Graecopithecus* (34, 19, 37-40). An upper premolar from Azmaka in Bulgaria was recently attributed to cf. *Graecopithecus* (19,41). Since the 1970's, many more fossils of thickly enameled late Miocene apes have been described, mostly from Macedonia (Northern Greece), but recently from Anatolia, attributed to the genus *Ouranopithecus* (Supplementary Figure 15). The affinities of *Ouranopithecus* have long been debated (5,7,9,10,18,19,37-51). The prevailing view until recently was that, regardless of taxonomic priority, *Graecopithecus* and *Ouranopithecus* were almost indistinguishable. However, re-analysis of the *Graecopithecus* mandible and premolar established with little doubt that these taxa are different (19,37).

*Taxonomic history of Ouranopithecus*

As the sample of late Miocene eastern Mediterranean hominids has expanded, interpretations of their taxonomy and phylogeny have changed. Traditionally, all samples apart from *Ankarapithecus* were attributed to *Ouranopithecus*. Complicating this interpretation is that *Graecopithecus* has priority over *Ouranopithecus*, the much better-known taxon. Some have suggested *Ouranopithecus* should be recognized as a junior subjective synonym of *Graecopithecus* (39,52). Others have suggested that the holotype of *Graecopithecus freybergi* is unsuitable due to its poor preservation (53). However, several researchers have noted important differences between the

northern (*Ouranopithecus*) and southern (*Graecopithecus*) Greek taxa, suggesting that they are correctly attributable to different genera (37-38). This latter interpretation has received strong support from the most recent analysis of the *Graecopithecus* specimen, which reveals several taxonomically and phylogenetically significant anatomical distinctions from *Ouranopithecus*, especially in subocclusal morphology, previously undescribed (19).

The evidence is strong for the recognition of two distinct genera of thickly enameled late Miocene apes in Greece, *Ouranopithecus* from the Vallesian (MN 10, 9.6-9.3 Ma) of Macedonia and *Graecopithecus* from the Turolian (MN 12, 7.2 Ma) of Attica (29,32,54). The holotype of *Ouranopithecus macedoniensis* (RPl 54) and the vast majority of the hypodigm come from the site of Ravin de la Pluie (9.6 Ma). The isolated partial cranium (XIR 1) from Xirochori, (9.3 Ma), which is geographically close to Ravin de la Pluie, is quite similar in morphology and is no doubt the same taxon. However, a matched set of jaws from Nikiti 1, about 130 km further to the southeast, are morphologically distinct from the Ravin de la Pluie/Xirochori samples and are also significantly younger, being dated to 8.5-9.0 Ma (32,54). It is possible that this sample does not belong to *Ouranopithecus macedoniensis* (47), but potentially to *Anadoluvius*. In addition to being close in age, the apes from Nikiti 1 and Çorakyerler share with *Graecopithecus* broad molars relative to corpus dimensions and more canine reduction than in *Ouranopithecus*. *Anadoluvius* shares premolar root reduction with *Graecopithecus* (not known for the Nikiti specimen) while the Nikiti mandible (NKT 21) shares a posteriorly positioned inferior transverse torus with *Graecopithecus* (not known for *Anadoluvius*.) The isolated upper premolar from Azmaka in Bulgaria is the same age as Pyrgos Vassilissis and more consistent morphologically with *Graecopithecus* than *Ouranopithecus* (29,19,41).

Given the results of this analysis we propose the following taxonomy of the taxa discussed in the text:

Supplementary Table 8: Taxonomy of hominoids discussed in the text[1].

- Hominoidea
  - Proconsulidae
    - *Ekembo*
    - *Proconsul*
    - *Samburupithecus*
  - Afropithecidae
    - *Afropithecus*
    - *Morotopithecus*
  - Hominidae
    - Griphopithecinae
      - *Griphopithecus*
      - *Equatorius*
      - *Nacholapithecus*
      - *Kenyapithecus*
    - Homininae
      - Dryopithecini
        - Dryopithecina
          - *Dryopithecus*
          - *Pierolapithecus*
          - *Anoiapithecus*
          - *Danuvius*
          - *Hispanopithecus*
          - *Rudapithecus*
        - Ouranopithecina
          - *Ouranopithecus*
          - *Graecopithecus*
          - *Anadoluvius*
      - Gorillini
        - *Gorilla*
      - Hominini
        - *Pan*
        - *Orrorin*
        - *Sahelanthropus*[2]
        - *Ardipithecus*
        - *Australopithecus*
        - *Paranthropus*

Ponginae

*Pongo*

*Sivapithecus*

*Ankarapithecus*

*Khoratpithecus*

Hominidae incertae cedis

*Chororapithecus*

*Nakalipithecus*

*Lufengpithecus*

Hominoidea incertae cedis

*Oreopithecus*

---

Notes: 1) Not all clades are named in this classification. There are at least two dryopithecin clades as well as separate *Pan and Ardipithecus/*australopithecine clades. Multiple clades are probably also contained within the Griphopithecinae and the Ponginae. 2) *Sahelanthropus* is never recovered as a hominin in our cladistic analyses but is nonetheless placed in that taxon here for two reasons. Hominin-like attributes of *Sahelanthropus* in the basicranium could not be included in this analysis as they are not preserved in any other fossil hypodigm except possibly *Ekembo*. In addition, as noted above, *Sahelanthropus* was coded from the literature. Coding criteria may not have been consistent with the criteria used in this analysis based on direct observation of original fossil material.

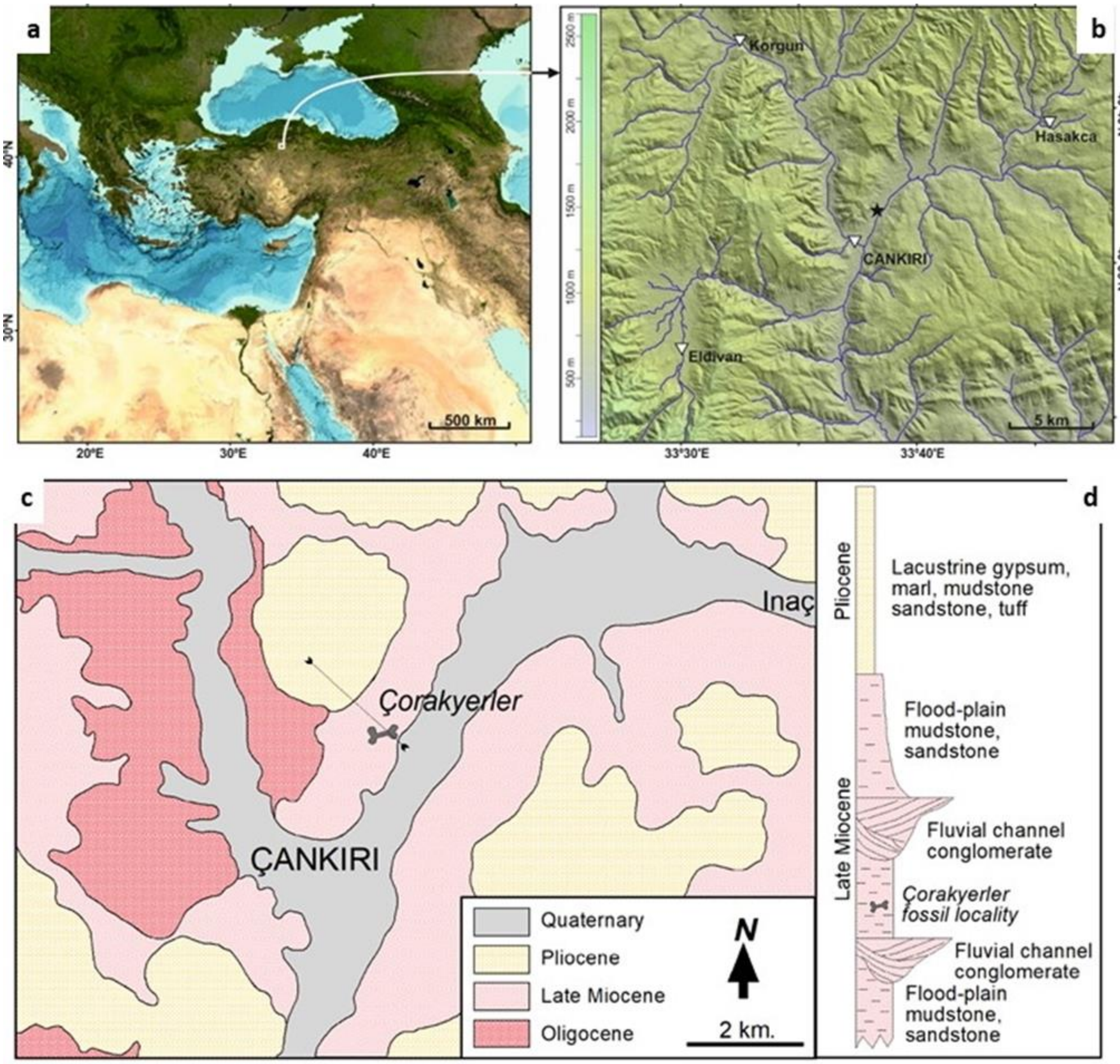


Supplementary Figure 16

(a) Regional location map of Anatolia and its surrounds (generated using the GEBCO-Sheaded Relief data in Global Mapper v19.0 software), (b) Close-up map of Çorakyerler fossil locality (generated using the SRTM-3arc-second resolution digital data in Global Mapper v19.0 software), (c) Geological map of the Çorakyerler fossil locality and its surrounds with section line indicated (59), (d) Stratigraphical log of the Çorakyerler fossil locality (not to scale).

The fossil locality of Çorakyerler (40°36'32"N, 33°38'01"E) is in the Çankırı Basin of the central Anatolian Cenozoic basin complex (55-60) (figure S 6.) The geological and geographic context of the locality is summarized in Supplementary Figure 16 (56-57). The basin-fill succession is composed of marine and terrestrial deposits of Paleogene to Neogene age. The Neogene of the basin is characterized by terrestrial sediments comprised of alluvial-fan, fluvial and lacustrine deposits. The Çorakyerler fossil locality is situated within the fluvial flood-plain deposits that were described lithostratigraphically as Tuğlu Formation by (56). A paleoenvironmental and chronological constraint of the unit has been determined by (55-58) based on paleontological constraints including micro and macro-mammal assemblages, ostracod, foraminifer, nannoplankton, pollen, palaemagnetism, isotope geochemistry, and major elemental compositions.

The "Lignite Deposit Exploration in Türkiye" project, conducted by a group of German and Turkish geologists, led to the discovery of Çorakyerler in the late 1960's. The first sampling of the site was carried out by O. Sickenberg and his team. The preliminary results, including an initial faunal list of 12 macromammalian taxa, were published (61). Relatively few publications are focused exclusively on the Sickenberg assemblage, which was said to be dominated by open country taxa dating broadly to the Turolian (51-64). Some studies mention Çorakyerler fossils in a comparative context but for the most part these researchers only had access to limited samples from the site (e.g. (64)). No fossil apes were recovered during this first phase of research. The second and current phase of research was initiated by the first author in 1997 (65). Through 2022 more than 3812 identifiable fossil specimens have been recovered including gnathic material from at least four hominine individuals.

The micromammals of Çorakyerler were described by (66). The micromammal assemblage is diverse, comprising twelve species. The assemblage is dominated by murines, with *Hansdebruijnia erksinae* accounting for over 50% of the fauna. A second murine, *Hansdebruijnia* cf. *H. neutrum*, is also present. *H. erksinae* was considered by the authors to be indicative of closed environments. The

eomyid *Keramidomys* also preferred more forested areas. Taxa from more open environments are numerous, but less represented. These include the gerbil *Pseudomeriones,* the cricetine *Allocricetus*, the porcupine *Hystrix,* the spalacid *Pliospalax* and the jerboa *Protalactaga*. The cricetine *Byzantinia*, also well represented in the assemblage, has more mixed preferences. (63) suggests a 'savannah-like' environment, which, based on the faunal list only, seems plausible. However, given the quantitative composition, the direct surroundings of Çorakyerler seem to have been more forested, in line with the assemblage of large mammals. In sum, the large mammal assemblage of Çorakyerler encompasses a mosaic ecosystem (Supplementary Table 11).

The large mammals of Çorakyerler were described partly by (64) (bovids) and (67) (proboscideans) while (65) provided a brief description of the materials collected from 1998 to 2001. More recent analysis has provided several additions to the fauna that include: carnivorans (*Indarctos atticus*, *Machairodus aphanistus*, *Promephitis*, *Parataxidea maraghana* and *Sivaonyx* nov.sp.), proboscidean (*Konobelodon*), chalicothere (*Ancylotherium pentelicum*), hipparionin horse (*Hipparion depereti*), suids (*Hippopotamodon* cf. *H. antiquus*) and giraffids (*Samotherium boissieri*) (68-73). Extremely high bovid biodiversity at Çorakyerler, including at least 10 species, has been recognized by the latest studies (74-75). These studies have also added two new Antilopinae, *Qurliqnoria corakensis* and *Gangraia anatolica* to the faunal list of Çorakyerler.

(55) bracket the age of the Çorakyerler locality between 8.11 and 7.64 Ma. This would imply, according to their figure 6, a correlation to the later part of MN 11, and suggests the survival of many Vallesian elements at Çorakyerler well into the Turolian, which seems an unlikely scenario. According to these authors, the proposed age bracket was the oldest option within MN 11 and MN 12 to fit the Çorakyerler magnetostratigraphic sequence, consisting of a long period of normal polarity followed by a short reversed chron. Presumably they did not consider a correlation to C4A, because this is older than the age they adopted for the MN 10/MN 11 boundary at 8.7 Ma as indicated by (58). The magnetostratigraphic analysis of (55) was carried out independently of the permitted project, leading to

some uncertainty in the provenance information provided in that study. It also relied heavily on the biostratigraphic results of (63), which included very little of the material recovered since 2000. Here, we consider a correlation with Chron C4n the most parsimonious fit between magnetostratigraphic and the latest biochronological studies (68-76). We therefore consider the fauna associated with the hominine best placed early in MN 11, about 8.7 Ma (Supplementary Figure 17).

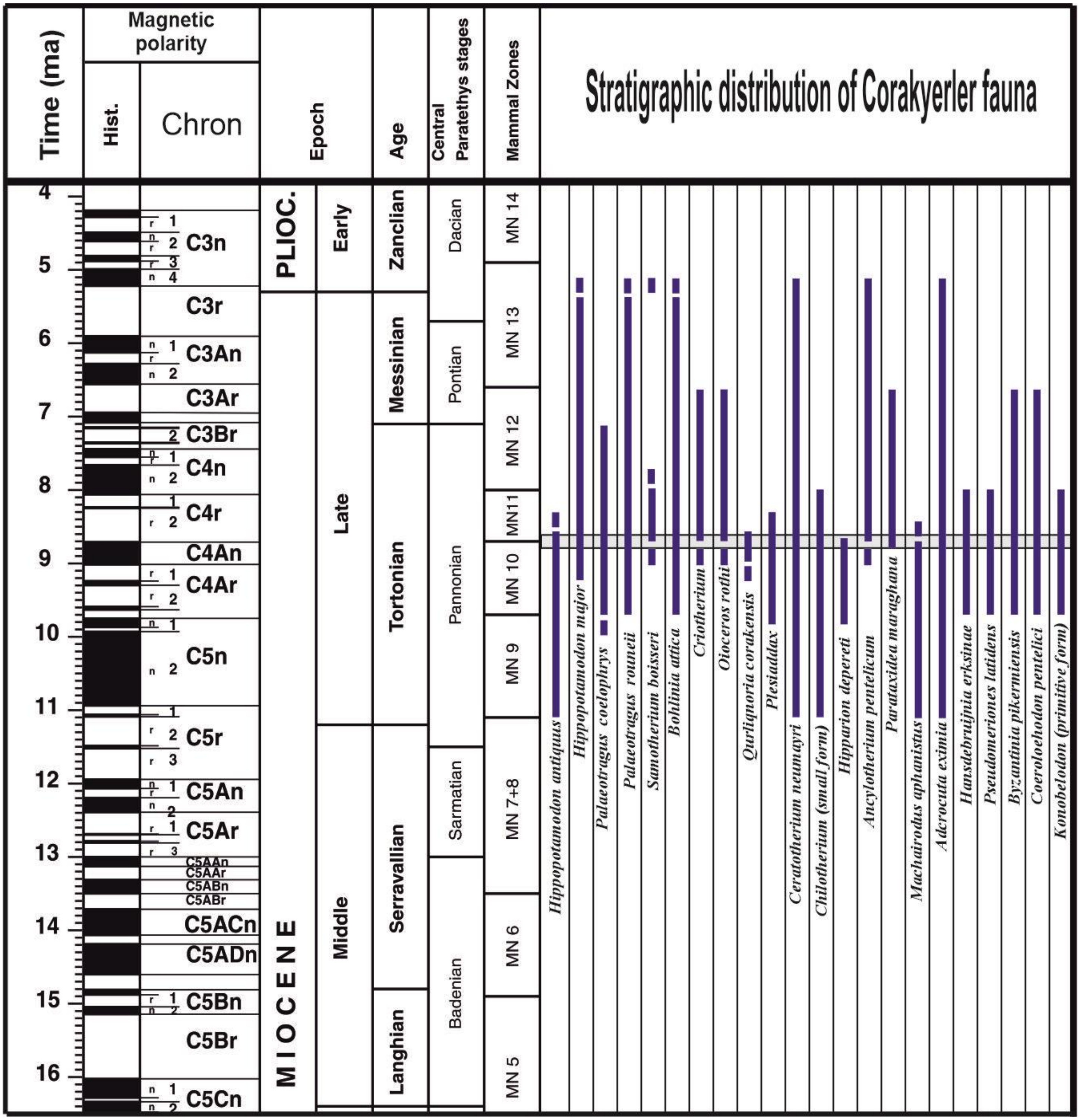


Supplementary Figure 17. Summary of the age determination for the Çorakyerler fauna.

**Supplementary Table 9. Faunal List**

**Artiodactyla**
**Suidae**
*Hippopotamodon major*
*Hippopotamodon* cf. *H. antiquus*

**Cervidae**
*Cervidae indet*

**Giraffidae**
*Palaeotragus cf. P. quadricornis*
*Palaeotragus cf. P. rouenii*
*Samotherium boissieri*
*Bohlinia attica*

**Bovidae**
*Sporadotragus* sp.
*Tragoportax gaudryi*?
*Prostrepsicerus* sp.
*Miotragocerus* (*Pikermicerus*) sp
*Gazella* sp.
*Oioceros* rothi
*Protoryx* sp.
*Plesiaddax inundates*
Ovibovini indet.
*Criotherium* sp.
*Qurliqnoria corakensis*
*Gangraia anatolica*

**Perissodactyla**
**Rhinocerotidae**
*Acerorhinus* sp.
*Chilotherium kowalevskii*
*Ceratotherium neumayri*

**Equidae**
*Hipparion dietrichi*
*Hipparion prostylum*
*Hipparion depereti*

**Chalicotheriidae**
*Ancylotherium pentelicum*

**Proboscidea**
**Gomphotheriidae**
*Choerolophodon pentelici*
*Konobelodon nov. s*p.

**Carnivora**
**Mustelidae**
*Sivaonyx nov*. sp.

*Plesiogulo nov*. sp.
*Parataxidea maraghana*

**Mephitidae**
*Promephitis* sp.

**Hyaenidae**
*Adcrocuta eximia*
*Chasmaporthetes* sp.
*Ictitherium* sp.

**Felidae**
*Machairodus aphanistus*

**Ursidae**
*Indarctos atticus*

**Primates**
*Anadoluvius turkae*

**Rodentia**
**Hystricidae**
*Hystrix* sp.

**Muridae**
**Murinae**
*Hansdebruijnia erksinae*
*Hansdebruijnia* cf. *H. neutrum*
**Cricetondontinae**
*Byzantinia pikermiensis*
*Byzantinia* aff. *B. hellenicus*
cf*. Rhinocerodon sp.*
**Cricetinae**
*Allocricetus aylasevimae*
**Gerbillinae**
*Pseudomeriones latidens*
**Spalacinae**
cf. *Pliospalax* sp. indet.
**Allactaginae**
*Protalactaga* aff. *P. major*

**Gliridae**
*Myomimus* sp.

**Eomyidae**
*Keramidomys* sp.

Supplementary Table 9. Fauna from Çorakyerler as of July 2022. Rodents from (66).

## Supplementary References


1. McNulty, K.P, Begun, D.R, Kelley, J., Manthi, F.M. & Mbua, E.N. A systematic revision of *Proconsul* with the description of a new genus of early Miocene hominoid. *J. Hum. Evol.* **84**, 42–61 (2015). https://doi.org/10.1016/j.jhevol.2015.03.009.

2. Andrews, P. A Revision of the Miocene Hominoidea from East Africa." Bull. Brit. Mus. Nat. Hist. (Geology) 30, no. 2: 85–224 (1978).

3. Nakatsukasa, M. and Kunimatsu, Y. *Nacholapithecus* and Its Importance for Understanding Hominoid Evolution. Evolutionary Anthropology: Issues, News, and Reviews 18 (3): 103–19 (2009) https://doi.org/10.1002/evan.20208.

4. McCrossin, M. L, and Benefit, B. R. On the Relationships and Adaptations of *Kenyapithecus*, a Large-Bodied Hominoid from the Middle Miocene of Eastern Africa. In Function, Phylogeny and Fossils: Miocene Hominoid Origins and Adaptations., edited by D. R Begun, C. V Ward, and M. D. Rose, 241–67. New York: Plenum Press, (1997).

5. Kunimatsu, Y. et al. A new Late Miocene great ape from Kenya and its implications for the origins of African great apes and humans. Proc. Natl Acad. Sci. 104, 19220-19225 (2007).

6. Bonis, L. de, Bouvrain, G. & Melentis, J. Nouveaux restes de primates hominoïdes dans le Vallésien de Macédoine (Grèce). C. R. Acad. Sci. D Paris 182, 379-382 (1975).

7. Bonis, L. de & Koufos, G. D. Our ancestors' ancestor: *Ouranopithecus* is a Greek link in human ancestry. Evol. Anthropol. 3, 75-83 (1994).

8. Kordos, L., and Begun. D.R. Primates from Rudabánya: Allocation of Specimens to Individuals, Sex and Age Categories. J. Hum. Evol. 40: 17–39 (2001).

9. Alba, D. M. Fossil apes from the Vallès-Penedès basin. Evol. Anthropol. 21, 254-269 (2012).

10. Begun, D. R. Fossil Record of Miocene Hominoids in Handbook of Paleoanthropology (eds. Henke, W. & Tattersall, I.) 1261-1332 (Springer 2015).

11. Kappelman, J. et al. Hominoidea (Primates) in Geology and Paleontology of the Miocene Sinap Formation, Turkey. (eds. Fortelius, M. et al.) 90-124 (Columbia University Press, (2003).

12. Begun, D.R. & Güleç, E. Restoration of the Type and Palate of *Ankarapithecus meteai*: Taxonomic, Phylogenetic, and Functional Implications. Am. J. Phys. Anthropol. 105, 279-314 (1998).

13. Kelley, J. The Hominoid Radiation in Asia, in: Hartwig, W. (Ed.), The Primate Fossil Record. Cambridge University Press., Cambridge, pp. 369-384. (2002.)

14. Kelley, J. Twenty-Five Years Contemplating Sivapithecus Taxonomy. In Interpreting the Past : Essays on Human, Primate, and Mammal Evolution in Honor of David Pilbeam, edited by David R. Pilbeam, Daniel Lieberman, Richard J. Smith, and Jay Kelley, 123–43. Boston ; Brill Academic Publishers (2005).

15. White, T. D., B. Asfaw, et al. Ardipithecus ramidus and the Paleobiology of Early Hominids. Science 326(5949): 64, 75-86. (2009).

16. Brunet, M. et al. A New Hominid from the Upper Miocene of Chad, Central Africa. Nature 418: 145–51 (2002).

17. Ishida, H. & Pickford, M. A new late Miocene hominoid from Kenya: Samburupithecus kiptalami gen. et sp. nov. C. R. Acad. Sci. Paris. 325, 823-829 (1997).

18. Suwa, G. et al. A new species of great ape from the late Miocene epoch in Ethiopia. Nature. 448, 921-924 (2007).

19. Fuss, J. et al. Potential hominin affinities of Graecopithecus from the Late Miocene of Europe. PLoS ONE 12 (e0177127) (2017).

20. Senut, B., et al. First Hominid from the Miocene (Lukeino Formation, Kenya). C. R. Acad. Sci., Sciences de La Terre et Des Planètes 332: 137–44 (2001).

21. Güleç, E. et al. A new great ape from the late Miocene of Turkey. Anthropol. Sci. 115, 153-158 (2007).

22. Bonis, L. d. & Koufos, G. The face and mandible of *Ouranopithecus macedoniensis*: description of new specimens and comparisons. J. Hum. Evol. 24: 469-491 (1993).

23. Begun, D. R. et al. Hominin origins: New evidence from the eastern Mediterranean. Am. J. Phys. Anthrop. 168, 15 (2019).

24. Emonet, E.G., Andossa, L., Taïsso Mackaye, H., Brunet, M., Subocclusal dental morphology of Sahelanthropus tchadensis and the evolution of teeth in hominins. Am. J. Phys. Anthropol. 153, 116-123 (2014).

25. Emonet, E.-G., Tafforeau, P., Chaimanee, Y., Guy, F., de Bonis, L., Koufos, G., Jaeger, J.-J., Three-dimensional analysis of mandibular dental root morphology in hominoids. Journal of Human Evolution 62, 146-154 (2012).

26. Smith, T. M., P. Tafforeau, et al. Enamel thickness and dental development in Rudapithecus hungaricus. J. Hum. Evol. 136: 102649. (2019).

27. Johanson, D. C., T. D. White, et al. Dental remains from the Hadar formation, Ethiopia: 1974–1977 collections. Am. J. of Phys. Anthropol. 57(4): 545-603. (1982).

28. Pugh, K. D. Phylogenetic analysis of Middle-Late Miocene apes. Journal of Human Evolution, 165, 103140. doi: https://doi.org/10.1016/j.jhevol.2021.103140 (2022).

29. Böhme, M. et al. Messinian age and savannah environment of the possible hominin *Graecopithecus* from Europe. PLoS ONE 12, e0177347 (2017).

30. Suwa, G. et al. The first hominoid from the Maragheh Formation, Iran. Palaeobiodiv. Palaeoenviron. 96, 373-381 (2016).

31. Agustí, J. et al. Late survival of dryopithecine hominoids in Southern Caucasus. J. Hum. Evol. 138, 102690 (2020).

32. Koufos, G. D. The Neogene mammal localities of Greece: faunas, chronology and biostratigraphy. Hell. J. Geosci. 41, 183-214 (2006).

33. Kappelman, J., A. Duncan, et al. Chronology. Geology and Paleontology of the Miocene Sinap Formation, Turkey. M. Fortelius, J. Kappelman, S. Sen and R. Bernor. New York, Columbia University Press: 41-66 (2003).

34. Freyberg, B. von, Die Pikermifauna von tour la Reine (Attica). Annales géologiques des Pays Helléniques, 3: p. 7–10 (1951).

35. Begun, D.R. & Güleç, E. Restoration of the Type and Palate of *Ankarapithecus meteai*: Taxonomic, Phylogenetic, and Functional Implications. Am. J. Phys. Anthropol. 105, 279-314 (1998).

36. Alpagut, B., et al. A new specimen of *Ankarapithecus meteai* from the Sinap Formation of central Anatolia. Nature 382, 349-351 (1996).

37. Begun, D. R. European Hominoids. In The Primate Fossil Record., edited by W. Hartwig, 339–68. Cambridge: Cambridge University Press (2002).

38. Koufos, G. D. & Bonis, L. de The Late Miocene hominoids *Ouranopithecus* and *Graecopithecus*. Implications about their relationships and taxonomy. Ann. Paleontol. 91, 227-240 (2005).

39. Martin, L. & Andrews, P. The phyletic positon of Graecopithecus freybergi KOENIGSWALD. Cour. Forsch. Inst. Sencken. 69, 25-40 (1984).

40. Koenigswald, G.H.R., Ein Unterkiefer eines fossilen Hominoiden aus dem Unterpliozän Griechenlands. Proc. Kon. Nederl. Akad. Wet. B, 75: p. 385-394 (1972).

41. Spassov, N. et al. A hominid tooth from Bulgaria: The last pre-human hominid of continental Europe. J. Hum. Evol 62, 138-145 (2012).

42. Begun, D.R. Nargolwalla, M.C., Kordos, L. European Miocene hominids and the origin of the African ape and human clade. Evol Anthropol 21, 10-23 (2012).

43. Begun, D. R. *Dryopithecus*, Darwin, de Bonis and the European origin of the African apes and human clade. Geodiversitas 31, 789–816 (2009).

44. Dean, D. & Delson, E. Second gorilla or third chimp? Nature 359, 676-677 (1992).

45. Nengo, I., Tafforeau, P., Gilbert, C.C., Fleagle, J.G., Miller, E.R., Feibel, C., Fox, D.L., Feinberg, J., Pugh, K.D., Berruyer, C., Mana, S., Engle, Z., Spoor, F. New infant cranium from the African Miocene sheds light on ape evolution. Nature 548, 169-174 (2017).

46. Bonis, L. de, Bouvrain, G. & Melentis, J. Nouveaux restes de primates hominoïdes dans le Vallésien de Macédoine (Grèce). C. R. Acad. Sci. D Paris 182, 379-382 (1975).

47. Bonis, Louis de, and George Koufos. The Phylogenetic and Functional Implications of Ouranopithecus Macedoniensis. In Function, Phylogeny, and Fossils: Miocene Hominoid Evolution and Adaptations, edited by David R. Begun, Carol V. Ward, and Michael D. Rose, 317–26. Boston, MA: Springer US, 1997. https://doi.org/10.1007/978-1-4899-0075-3_15.

48. Finarelli, J. A. & Clyde, W.C. Reassessing hominoid phylogeny: evaluating congruence the morphological and temporal data. Paleobiology. 30, 614-651(2004).

49. Folinsbee, K. & Brooks, D. Miocene hominoid biogeography: Pulses of dispersal and differentiation. J. Biogeog. 34, 383-397 (2007).

50. Young, N. M. & MacLatchy, L. The phylogenetic position of *Morotopithecus*. J. Hum. Evol. 46, 163-184 (2004).

51. Ioannidou, M. et al. A new three-dimensional geometric morphometrics analysis of the *Ouranopithecus macedoniensis* cranium (Late Miocene, Central Macedonia, Greece). Am. J. Phys. Anthropol. 170, 295-307 (2019).

52. Cameron, D. W. The taxonomic status of *Graecopithecus*. Primates. 38, 293-302 (1997).

53. Groves, C. P. A Theory of Primate and Human Evolution. (Clarendon Press, 1989).

54. Koufos, G. D. History, stratigraphy and fossiliferous sites. Geobios. 49, 3-10 (2016).

55. Kaya, F. et al. Magnetostratigraphy and paleoecology of the hominid-bearing locality Çorakyerler, Tuglu Formation (Çankiri Basin, Central Anatolia). J. Vert. Paleontol. 36, DOI: 10.1080/02724634.2015.1071710 (2016).

56. Kaymakçı, N. et al. Neogene tectonic development of the Çankırı basin (central Anatolia, Türkiye). TPJD Bülteni. 13, 27-56 (2001).

57. Mazzini, I. et al. Palaeoenvironmental and chronological constraints on the Tuğlu Formation (Çankırı Basin, Central Anatolia, Türkiye). Turk. J. Earth Sci. 22, 747-777 (2013).

58. Şen, Ş. et al. Mammalian biochronolgy of Neogene deposits and its correlation with the lithostratigraphy in the Çankırı-Çorum basin, central Anatolia, Türkiye. Eclog. Geolog. Helv. 91, 307-320 (1998).

59. Sevin, M. & Uğuz, M.F. 1/100.000 scaled geological map sheets of Türkiye (Çankırı- G31). Mineral Research and Exploration Directorate of Türkiye: Ankara (2011).

60. Öcal H., Turhan N. & Göktaş F. Geological maps of Türkiye in 1:100000 scale: Çankırı G31 sheet. Mineral Research and Exploration Directorate of Türkiye, Ankara 147, 32 pp. (2011).

61. Sickenberg, O. et al. Die Gliederund des höheren Jungertertiärs und Altquartärs in der Türkei nach Vertebraten und ihre Bedeutung für die internationale Neogen-Stratigraphie. Geol. Jb. B(15), 1-167 (1975).

62. Heissig, K. Rhinocerotidae aus dem Jungtertiar Anatoliens. Geol. Jb. B(15), 145- 151 (1975).

63. Geraads, D. Large Mammals from the late Miocene of Corakyerler, Cankiri, Türkiye. Acta Zoolog Bulgar. 65, 381-390 (2013).

64. Köhler, M. Funktionsmorphologie des Skelettbaus von Pecora (Mammalia) und ihre Anwendung zur Interpretation der Paläokologie des Neogens. Paleontol. Evol. (1990).

65. Sevim, A. and Y. Kiper, 1999 Yılı Çankırı Çorakyerler Kazısı, 22. Kazı Sonuçları Toplantısı, (ed Basımevi, K.B.M.K.) 37-46 (Ankara, 2001).

66. Ünay, E., Bruijn, H. de & Suata-Alpaslan, F. Rodents from the upper Miocene hominoid locality Çorakyerler (Anatolia). Beitr. Paläont. 30, 463–467 (2006).

67. Gaziry, A.W. Jungtertiare Mastodonten aus Anatolien (Türkei). Geolog. Jahrb. B. 22, 1–143 (1976).

68. Mayda, S., Sevim Erol, A. & Yavuz, A. Y. Carnivora from Çankırı-Corakyerler Hominoid Locality. 68th Geological Congress of Türkiye, 06-10 Nisan/April 2015. 489 (2015).

69. Sevim Erol, A., Yavuz, A. Y. & Mayda, S. Çorakyerler Locality- The Center of the Youngest Hominoids of Anatolia. in International Congress of Anthropological Sciences (ICAS), 9th-11th April 2015. Ankara, Türkiye (2015).
70. Sevim Erol, A. et al. Çorakyerler Kazısı 2017 Yılı. 40. (ed Toplantısı, U. K. S.) 311-324 (2018).
71. Sevim Erol, A. et al. Çorakyerler Kazısı 2016 Yılı. 39. (ed Toplantısı, U. K. S.) 521-535 (2017).
72. Tarhan, E. et al. Zürih Üniversitesi Paleontoloji Müzesi'nde Bulunan Çorakyerler Suidae Materyallerinin Revizyonu. MASROP E-Dergi 2018 12.2, 58-69 (2012).
73. Yavuz, A. Y. et al. Çorakyerler Lokalitesi Hystricidae Buluntuları. MASROP E-Dergi 2018 12.2, 70-75 (2018).
74. Kostopoulos, D. et al. Qurliqnoria (Bovidae, Mammalia) from the Upper Miocene of Çorakyerler (Central Anatolia, Türkiye) and its biogeographic implications. Palaeoworld. doi.org/10.1016/j.palwor.2019.10.003 (2019).
75. Kostopoulos, D. et al. A new late Miocene bovid (Mammalia: Artiodactyla: Bovidae) from Çorakyerler (Türkiye). Fossil Record. doi.org/10.5194/fr-24-9-2021
76. Agusti, J. et al. A calibrated mammal scale for the Neogene of Western Europe. State of the art. Earth-Sci. Rev. 52, 247-260 (2001).
