# Supplementary material for: A new ape from Türkiye and the radiation of late Miocene hominines
Source: Commun Biol. 2023 Aug 23;6:842. doi: 10.1038/s42003-023-05210-5 (PMC10447513; doi:10.1038/s42003-023-05210-5)
Supplement: Supplementary file 3 — Description of Additional Supplementary Files [file 42003_2023_5210_MOESM3_ESM.pdf]

Description of Additional Supplementary Files

File name: Supplementary Data 1
Description: Principle components analysis

File name: Supplementary Data 2
Description: Taxon character states

File name: Supplementary Data 3
Description: Nexus file
