# Supplementary material for: A new ape from Türkiye and the radiation of late Miocene hominines
Source: Commun Biol. 2023 Aug 23;6:842. doi: 10.1038/s42003-023-05210-5 (PMC10447513; doi:10.1038/s42003-023-05210-5)
Supplement: Supplementary file 7 — Reporting Summary [file 42003_2023_5210_MOESM7_ESM.pdf]

nature portfolio

Corresponding author(s): David R. Begun and Ayla Sevim-Erol

Last updated by author(s): Jul 20, 2023

Reporting Summary

Nature Portfolio wishes to improve the reproducibility of the work that we publish. This form provides structure for consistency and transparency in reporting. For further information on Nature Portfolio policies, see our Editorial Policies and the Editorial Policy Checklist.

Statistics

For all statistical analyses, confirm that the following items are present in the figure legend, table legend, main text, or Methods section.

n/a	Confirmed	
☐	☒	The exact sample size (*n*) for each experimental group/condition, given as a discrete number and unit of measurement
☐	☒	A statement on whether measurements were taken from distinct samples or whether the same sample was measured repeatedly
☐	☒	The statistical test(s) used AND whether they are one- or two-sided<br>*Only common tests should be described solely by name; describe more complex techniques in the Methods section.*
☐	☒	A description of all covariates tested
☐	☒	A description of any assumptions or corrections, such as tests of normality and adjustment for multiple comparisons
☐	☒	A full description of the statistical parameters including central tendency (e.g. means) or other basic estimates (e.g. regression coefficient) AND variation (e.g. standard deviation) or associated estimates of uncertainty (e.g. confidence intervals)
☐	☒	For null hypothesis testing, the test statistic (e.g. *F*, *t*, *r*) with confidence intervals, effect sizes, degrees of freedom and *P* value noted<br>*Give P values as exact values whenever suitable.*
☒	☐	For Bayesian analysis, information on the choice of priors and Markov chain Monte Carlo settings
☒	☐	For hierarchical and complex designs, identification of the appropriate level for tests and full reporting of outcomes
☒	☐	Estimates of effect sizes (e.g. Cohen's *d*, Pearson's *r*), indicating how they were calculated

Our web collection on statistics for biologists contains articles on many of the points above.

Software and code

Policy information about availability of computer code

Data collection	n/a
Data analysis	PAST, EXCEL, TNT, MESQUITE, AMIRA

For manuscripts utilizing custom algorithms or software that are central to the research but not yet described in published literature, software must be made available to editors and reviewers. We strongly encourage code deposition in a community repository (e.g. GitHub). See the Nature Portfolio guidelines for submitting code & software for further information.

Data

Policy information about availability of data

All manuscripts must include a data availability statement. This statement should provide the following information, where applicable:

- Accession codes, unique identifiers, or web links for publicly available datasets
- A description of any restrictions on data availability
- For clinical datasets or third party data, please ensure that the statement adheres to our policy

All data generated or analyzed during this study are included in this publication (and Supplementary Materials). The computed tomography scans are available from the corresponding author on reasonable request. The new taxon has the following Life Science Identifier: urn:lsid:zoobank.org:act:FE6E46C7-BA39-428C-88E7-DC5FB2F5BE43

Human research participants

Policy information about studies involving human research participants and Sex and Gender in Research.

Reporting on sex and gender	n/a
Population characteristics	n/a
Recruitment	n/a
Ethics oversight	n/a

Note that full information on the approval of the study protocol must also be provided in the manuscript.

Field-specific reporting

Please select the one below that is the best fit for your research. If you are not sure, read the appropriate sections before making your selection.

☐ Life sciences ☐ Behavioural & social sciences ☒ Ecological, evolutionary & environmental sciences

For a reference copy of the document with all sections, see nature.com/documents/nr-reporting-summary-flat.pdf

Ecological, evolutionary & environmental sciences study design

All studies must disclose on these points even when the disclosure is negative.

Study description	Description and analysis of a new genus of Miocene ape from Turkey.
Research sample	Fossil apes from the Miocene of Europe, Anatolia, Africa and Asia; samples of extant ape skeletons from repositories in the US and Europe.
Sampling strategy	Sample sizes for fossils based on availability; samples sizes for extant samples always exceed 10 and usually over 50.
Data collection	Data collected by the second author using calipers. CT scan data collected by author six.
Timing and spatial scale	n/a
Data exclusions	no data excluded
Reproducibility	All original raw measurements of fossil provided in tables. Phylogenetic data matrix provided in a format "ready-to-use"
Randomization	Groups according to established taxonomy
Blinding	n/a

Did the study involve field work? ☐ Yes ☒ No

Reporting for specific materials, systems and methods

We require information from authors about some types of materials, experimental systems and methods used in many studies. Here, indicate whether each material, system or method listed is relevant to your study. If you are not sure if a list item applies to your research, read the appropriate section before selecting a response.

Materials & experimental systems

n/a	Involved in the study
☒	☐ Antibodies
☒	☐ Eukaryotic cell lines
☐	☒ Palaeontology and archaeology
☒	☐ Animals and other organisms
☒	☐ Clinical data
☒	☐ Dual use research of concern

Methods

n/a	Involved in the study
☒	☐ ChIP-seq
☒	☐ Flow cytometry
☒	☐ MRI-based neuroimaging

Palaeontology and Archaeology

Specimen provenance: Çorakyerler (40°36'32"N, 33°38'01"E) is in the Çankırı Basin of the central Anatolia

Specimen deposition: Specimens deposited in the Department of Anthropology, Ankara University

Dating methods: New dates based on a revised biochronology and a reinterpretation of the magnetostratigraphy.

☒ Tick this box to confirm that the raw and calibrated dates are available in the paper or in Supplementary Information.

Ethics oversight: Permit E-94949537-160.01.01-3883433, MINISTRY OF CULTURE AND TOURISM
General Directorate of Cultural Heritage and Museums. PI Dr. Ayla Sevim Erol

Note that full information on the approval of the study protocol must also be provided in the manuscript.
